# Time Restricted Eating: A Valuable Alternative to Calorie Restriction for Addressing Obesity?

**DOI:** 10.1007/s13679-025-00609-z

**Published:** 2025-02-03

**Authors:** Maria Eugenia Parrotta, Luca Colangeli, Valeria Scipione, Carolina Vitale, Paolo Sbraccia, Valeria Guglielmi

**Affiliations:** 1 Department of Systems Medicine, University of Rome Tor Vergata, Rome, Italy; 2 Internal Medicine Unit - Obesity Center, University Hospital Policlinico Tor Vergata, Rome, Italy

**Keywords:** Time restricted eating, Circadian rhythm, Biological clock, Obesity, Appetite

## Abstract

**Purpose of Review:**

In this review, we summarize the molecular effects of time-restricted eating (TRE) and its possible role in appetite regulation. We also discuss the potential clinical benefits of TRE in obesity.

**Recent Findings:**

TRE is an emerging dietary approach consisting in limiting food intake to a specific window of time each day. The rationale behind this strategy is to restore the circadian misalignment, commonly seen in obesity. Preclinical studies have shown that restricting food intake only during the active phase of the day can positively influence several cellular functions including senescence, mitochondrial activity, inflammation, autophagy and nutrients’ sensing pathways. Furthermore, TRE may play a role by modulating appetite and satiety hormones, though further research is needed to clarify its exact mechanisms. Clinical trials involving patients with obesity or type 2 diabetes suggest that TRE can be effective for weight loss, but its broader effects on improving other clinical outcomes, such as cardiovascular risk factors, remain less certain.

**Summary:**

The epidemic proportions of obesity cause urgency to find dietary, pharmacological and surgical interventions that can be effective in the medium and long term. According to its molecular effects, TRE can be an interesting alternative to caloric restriction in the treatment of obesity, but the considerable variability across clinical trials regarding population, intervention, and follow-up duration makes it difficult to reach definitive conclusions.

**Supplementary Information:**

The online version contains supplementary material available at 10.1007/s13679-025-00609-z.

## Introduction

The epidemic incidence of obesity and overweight causes urgency to find dietary, pharmacological and surgical interventions that can be effective [1]. The guidelines for the management of overweight and obesity in adults recommend reducing calories intake by the 25–30% of the total daily intake (1200–1800 kcals/day) [2], but weight loss is almost inevitably followed by weight regain. In fact, the organism physiologically reacts to energy restriction increasing appetite, reducing satiety and reducing energy expenditure [3–8]. This physiological compensation results from millennials of evolutionary adaptations to food scarcity [9, 10]. Hence, dietary strategies other than calorie restriction are necessary [11]. Time restricted eating (TRE) emerges as a new option, supported by an increasing body of evidence that defines its safety and therapeutic potential [12]. Moreover, interesting molecular findings highlight that fasting-feeding cycles positively interfere with some cellular process involved in metabolic functions, cell survival, inflammatory and mitochondrial processes [13, 14]. Furthermore, TRE seems to counterattack the circadian misalignment involved in the development of some metabolic alterations in obesity [15]. In fact, beyond weight loss, therapeutic programs should consider the multisystemic face of obesity and the chronic and progressive natural history of this disease [16].

In this review, we summarize the mechanisms that make TRE a viable therapeutic option for treating obesity. Its influence on the biological clock can impact intracellular mechanisms and hormones secretion, improving metabolic signaling and finally contributing to weight loss and to better control of cardiovascular risk factors in patients affected by obesity [15].

## Time Restricted Eating: Not What, But When

### Circadian Rhythm, Master Circadian Pacemaker and Peripheral Clocks

Nearly all living organisms, from single-celled cyanobacteria to mammals, developed a system to guarantee that physiological functions take place at appropriate times during the night or the day and to adapt the activity/rest cycle to energy intake and expenditure [17, 18]. The circadian clock mechanisms evolved to enable organisms to anticipate recurrent and periodical changes in their environment, so that biological processes can be stimulated or inhibited in advance as needed to benefit the organism [15, 17]. For example, DNA replication may be inhibited during UV radiation exposure to prevent DNA mutations, and food consumption and energy expenditure may be synchronized by the sleep–wake rhythm [19, 20].

In mammals, this system functions as an endogenous clock, which consists of a master clock located in the brain and secondary clocks located in the peripheral tissues. The central clock influences the peripheral clocks in a hierarchal chain [21] and both these systems regulate a cycle that is called circadian rhythm.

Circadian rhythm happens throughout the 24-h and entails modifications in biology and behavior, ranging from visible changes in activities, like the sleep–wake and fasting-feeding cycles, to more subtle and involuntary rhythms such as blood pressure oscillation, body temperature regulation, hormone blood release and many other metabolic processes [19]. Evidence suggests that dysfunction in these rhythms contributes to ageing and to the development of chronic diseases [22–24].

The master circadian clock is situated in the hypothalamic suprachiasmatic nucleus (SCN) [25] and it is regulated by changes in the environment, called *zeitgebers* [19]. The principal *zeitgeber* of the SCN is the change in light–dark cues [21], that is detected by the melanopsin-containing ganglion cells in the retina [15]. Regarding peripheral clocks, it seems that food consumption is the main *zeitgeber* [14], as mealtimes can directly affect the activity of peripheral clocks located in liver and gut [21].

Circadian clocks are present in every single cell of the human body and function in a self-regulated manner oscillating autonomously, following an endogenous period of 24 h, relying on a network of transcription-translation feedback [21, 25]. This 24-h cycle functions thanks to autoregulatory transcriptional feedback, depending on the rhythmic expression of clock-controlled genes [25, 26]. Transcriptional activators of the cellular clocks include the circadian locomotor output cycles kaput (CLOCK) [25] and brain and muscles ARNT-like1 (BMAL1) proteins [25, 27]. CLOCK and BMAL1 form a heterodimer (CLOCK:BMAL1) that positively regulates the expression of clock-controlled genes (CCGs) and the transcription of its self-repressors, the transcriptional repressor period 1 and 2 (PER1 and PER2, respectively) and cryptochrome 1 and 2 (CRY1 and CRY2). PER and CRY accumulate in the cytoplasm over time, forming a complex that migrates into the nucleus and inhibits the CLOCK:BMAL1 heterodimer. Thus, this system works as negative feedback with delay [28]. The complex CLOCK:BMAL1 stimulates the transcription of the retinoic acid receptor-related orphan receptor (ROR), and the nuclear hormone receptors REV-ERB; both ROR and REV-ERB coordinate CCGs, regulating their cyclical oscillation [29].

Although both the central and peripheral clocks are based on the same CCGs network, their dominating *zeitgebers* are dark–light changes and food consumption, respectively [29].

However, experiments on animal models demonstrated that rodents exposed to daytime feeding or nighttime feeding developed different effects on both central and peripheral circadian clocks [30], while mice exposed to nighttime feeding or freely fed uncoupled the central and secondary clocks synchronization compared to daytime fed mice [30]. Moreover, according to Stokkan et al., feeding cycles can regulate hepatic circadian clock even in SCN lesioned mice [31]. So, even though the central clock appears to regulate the peripheral clocks in a hierarchical way [21], changing a peripheral *zeitgeber* such as meal timing can actually have an effect on the central clock.

### Definition of Time Restricted Eating

The term fasting indicates the abstinence from food and beverages over a defined period of time [32]. Fasting has been practiced for millennia for hygienic and religious reasons, which were related to food preservation capabilities and seasonal access to fruits, leaves and grains [15, 33]. Various religions have been regulating food intake during certain times of the year, such as the Muslim Ramadan [34, 35] and the catholic Lent [36].

Intermittent fasting is an umbrella term referring to various dietary therapies that restrict the timing of meal intake rather than the content [15]. The most popular modalities of fasting are 1) alternate-day fasting which consists in the alternation of days with and without caloric intake; 2) alternate-day modified fasting, that alternates days without calorie restriction and days with a caloric intake limited to 25% of daily energy requirement; 3) period fasting, which consists of ad libitum eating days mixed with caloric restriction days over a one week long cycle; 4) TRE [15], a dietary approach in which food consumption is limited in a specific time window during the day (from 4 to 12 h) without caloric restriction [14]. Therefore, TRE stands out from the other kinds of intermittent fasting regimes because it does not need calories counting during the day [14].

Recently, fasting regimes gained attention as an alternative to caloric restriction regimes due to their potential to improve metabolic health by eliciting adaptative changes such as lowering basal metabolic rates, triggering ketogenesis and lipolysis, lowering oxidative stress, and influencing hormones’ hematic concentration [15, 32, 37]. Data found that intermittent fasting attempts to improve metabolic health by leveraging food intake with circadian physiology [15]. Particularly, most research implies that the advantages of TRE, including greater insulin sensitivity, may depend on aligning the fasting-feeding cycle with the circadian rhythm [14].

### Effects of TRE on Peripheral Tissues Circadian Clocks

Time restricted feeding (TRF) was initially studied in rodents to understand food anticipatory activity and behaviors, leading to the discovery that food providing is the main *zeitgeber* for the peripheral circadian clock, particularly in the liver [38, 39]. Accordingly, TRE may influence the expression of circadian clock related genes in peripheral organs such as adipose tissue, gut, muscles and liver itself [14, 15].

#### Liver

TRE may have a significant impact on hepatic circadian clock, as its expression of CCGs is modulated by mealtimes [40]. According to Vollmers et al., food intake and temporal pattern of feeding determined the amplitude and the phase of circadian transcriptome in wild type mice’ liver, compared to CRY1 and CRY2 knock-out mice [41]. TRF can induce metabolic rhythms also in liver-specific BMAL1 and REV-ERB knock-out mice [42]. Furthermore, fasting can induce the expression of agouti-related peptide [43, 44], which triggers liver rhythm-related transcriptional factors, such as BMAL1, CLOCK, PER and CRY. [43] Hatori et al. demonstrated an increase in both adenosine monophosphate (AMP) concentrations and AMP-activated protein kinase (AMPK) activity in the liver of rodents exposed to a 18-weeks long TRF regimen [45]. AMPK, activated by fasting, controls the expression and degradation of a lot of circadian proteins, such as CRY1, CRY2, PER and CLOCK:BMAL [46–48]. On the other hand, also mammalian target of rapamycin complex (mTORC1), which is activated by feeding, interacts with several circadian clock proteins [49, 50]. It is interesting, though, that AMPK and mTOR mutually inhibit [51].

Evidence suggests that TRE can regulate also the rhythmicity of hepatic cAMP-response element binding protein (CREB)c, enhancing the interaction between CREB and its coactivator CREB-regulated transcription coactivator 1 (CRTC1). Thus, it promotes the binding of the complex CREB-CRTC1 with the histone acetyltransferase CREB binding protein (CBP) [52]. By its side, the CREB-CRTC1-CBP complex stimulates hepatic clock genes *PER1* and *PER2* [52], while the CRY-cAMP-CREB axis regulates gluconeogenesis [41, 53].

#### Skeletal Muscle

Data on adult mice describe that fasting can affect circadian clock in skeletal muscle directly by modulating tissues clock genes (*BMAL1* and *PER*) and indirectly by modulating some clock genes regulators including ribosomal protein S6 and tripartite motif containing 63 *(TRIM63 or MuRF1*) [54]. In skeletal muscles the activity of AKT/mTOR pathway promotes protein synthesis by phosphorylating two major targets, S6 kinase 1 (S6K1) and eukaryotic initiation factor 4E-binding protein 1 (4E-BP1) [55]. Phosphorylating activity of AKT/mTOR exhibits temporal regulation in fasting animals, as period of fasting results in large decreases in phosphorylation of both AKT and S6K1 in muscles, indicating inactivation [56]. On the other hand, mTOR, stimulated by feeding, increases the availability of insulin and amino acids and regulates glucose and protein metabolism [56].

Studies on Drosophila and mice found that obesity can disrupt skeletal muscles’ circadian clock and function, and TRF may play a role in preventing muscle disruption and maintaining circadian rhythm in muscles mitochondrial respiration [56]. On the other hand, mice fed with a TRF regimen during inactive phase the diurnal regularity of mitochondrial respiration in skeletal muscles was disrupted [56].

#### Adipose Tissue

Also, white adipose tissue clock regulators may be affected by TRE. Several of adipose tissue’s functions cyclically oscillate, particularly the expression of fibroblast growth factor 21 (FGF21) [57] and hormone sensitive lipase (HSL) [57, 58]. TRF can prevent the disruption of FGF21 circadian oscillation secondary to a high fat diet [57]. Matoba et al. linked fasting to the secretion of Kruppel-like factor 15 (KLF15), which is capable of adapting lipid metabolism to nutrient availability through the promotion of lipolysis during fasting and lipid synthesis during feeding [59]. An interesting study showed that fasting can modulate the transcription of circadian clock genes in human adipose tissue, independent of the time of day, corroborating the hypotheses that meal timing can improve metabolism regulation [60].

#### Gut

Regarding the effects of TRE on gut, Brooks et al. described how the intestinal microbiota coordinates a rhythmic innate immune response that is linked to meal timing in order to anticipate gut exposure to microbes [61]. Circadian clock tailored by food intake rhythms and subsequent epithelial attachment by segmented filamentous bacteria (SFB) coordinates the expression of antimicrobial protein and the activation of an immunological circuit involving group 3 innate lymphoid cells. STAT3 itself regulates immune response through the expression of antimicrobial proteins such as regenerating islet‐derived protein 3*γ* (REG3G), lipocalin‐2 (LCN2) and S100A8 [61]. These oscillations have been found to be perturbed by a high fat diet [62] and to be restored by TRF [62–65].

### Effect of TRE on Circadian Misalignment

Studies have shown that TRE may have a noteworthy benefit on metabolic dysregulation in people with circadian misalignment [15]. Circadian misalignment is described as dysregulated cyclical recurrent behaviors, such as sleeping/activity and eating/fasting, during the 24-h cycle. This misalignment can alter metabolic homeostasis [66] and increases the risk of metabolic, renal and cardiovascular disease [67–69]. Evidence from knockout mice models clarify the role of the circadian clock in metabolic homeostasis. For example, Rudic et al. demonstrated that the inactivation of BMAL1 and CLOCK suppresses the 24-h variation in glucose and triglycerides concentration in circulating blood, depresses gluconeogenesis after insulin-induced hypoglycemia, but does not impact the counterregulatory response of glucagon and corticosterone [70]. This knowledge comes not only from mice but also from observational studies in people working in non-standard shifts and from simulated shift work or forced desynchrony experimental protocols [15]. Evidence agrees that circadian misalignment causes insulin resistance, decreases oral glucose tolerance and increases sensitivity to DNA damage [71–73]. Intermittent fasting regimens have shown, both in rodents’ and humans’, the ability to reduce circadian misalignment [15]. Particularly, TRE prevents the increase in adiposity and body weight, and attenuates glucose intolerance, β-cells dysfunction and consequent circadian misalignment triggered by shift work [74, 75].

## Fasting-Feeding Cycles Effects on Cellular Mechanisms

**Fig. 1:**
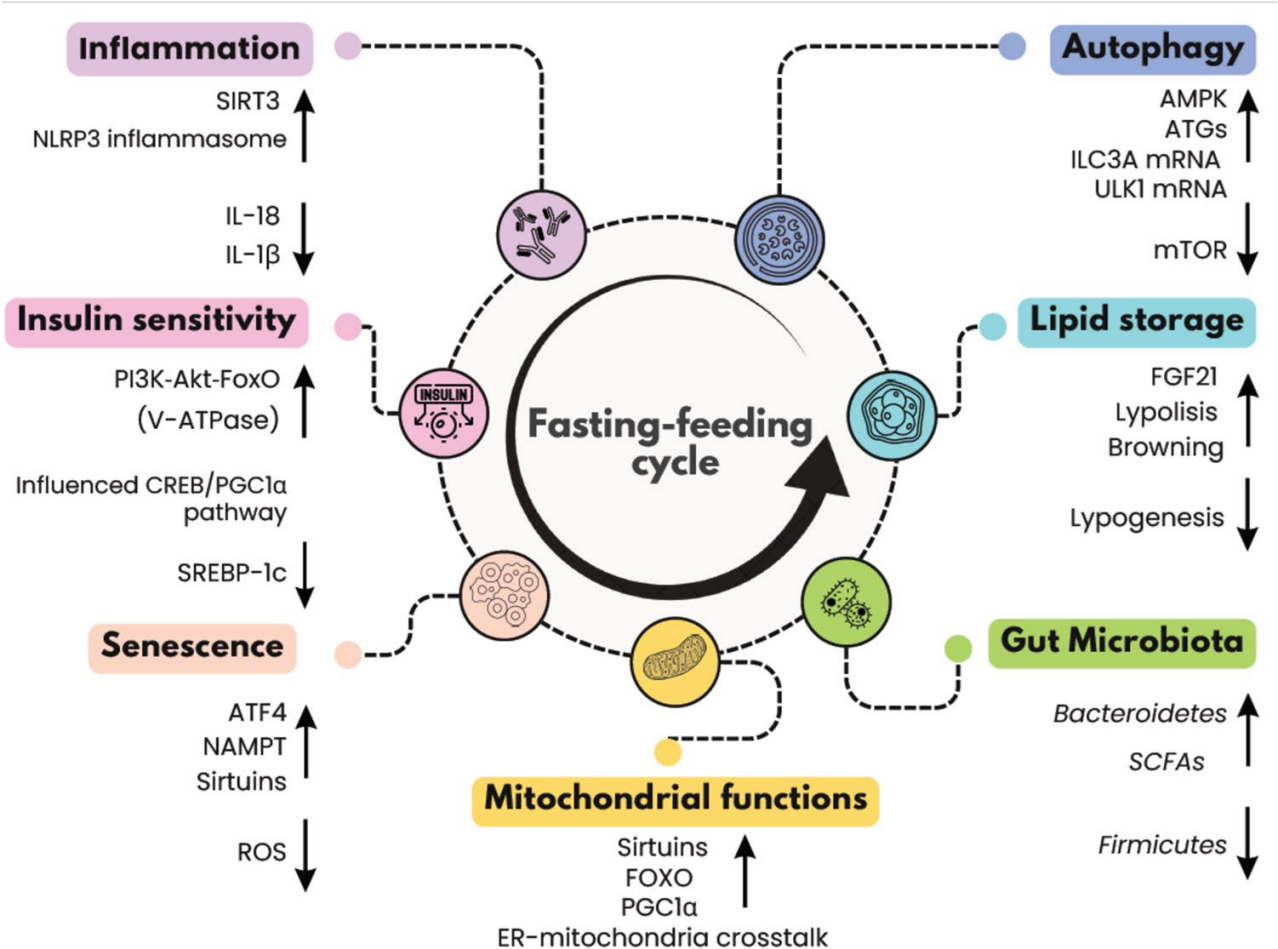
Fasting-feeding cycle affects several cellular mechanisms and influences gut microbiota. Abbreviations: *ER*, endoplasmic reticulum; *ROS*: reactive oxygen species; *SCFAs*: short chain fatty acids

Several molecular pathways appear to be influenced by the timing of feeding, not only metabolic signaling but also mechanisms involved in mitochondrial functions, cellular senescence, autophagy and inflammatory response [13] (Fig. 1). Dysregulation in these pathways can lead to the development of several chronic diseases such as cancer, neurological disease, and metabolic dysfunctions such as insulin resistance and dyslipidemia [14, 15, 76].

### Autophagy

Autophagy refers to the variety of intracellular homeostatic and protective processes that activate in order to provide energy to cells, when nutrients are insufficient. This process happens through the formation of autophagosomes. Autophagosomes incorporate organelle or intracytoplasmic matter and fuse with lysosomes for their enzymatic degradation [77]. The formation of autophagosomes is mediated by a complex pathway that involves many molecules, overall, the autophagy-related (ATG) family proteins [78]. Starvation seems to trigger autophagy directly inducing deacetylation of ATG4B [79]. Furthermore, AMPK and mTOR—activated respectively by fasting and feeding—work together to control autophagy. In fact, AMPK can directly trigger autophagy by phosphorylating the Unc-51-like kinase 1 (ULK1, an ortholog of ATG1) [80], activating the pro-autophagy vacuolar protein sorting 34 (VPS34) complex (which works with the ATG members proteins) [81], and indirectly by inhibiting mTOR. mTOR can inhibit autophagy by phosphorylation of ULK1, ATG13 and ATG14L in the VPS34 complex [51, 82]. Also, mTOR inhibits the transcription factor EB (TFEB), a transcriptional factor of lysosomal and autophagy genes [83]. ATG1 and ATG8a are expressed following circadian rhythms and studies showed that TRF upregulates their expression through CCGs [84, 85]. In humans, TRE seems to enhance expression of *LC3A* gene, which encodes for another structural component of the autophagosomes’ membranes [86]. Moreover, TRE enhances the expression of ULK1 by increasing the levels of miRNA involved in mTOR signaling pathway [87]. The relationship between obesity and autophagy is complex and not completely understood [88]. Even if there is limited evidence about TRE effects on autophagy in humans, it seems that fast-feed cycle can re-establish the physiological interplay between all the protagonists of this important homeostatic mechanism.

### Mitochondrial Dysfunction

Mitochondrial dysfunction refers to the incapacity of mitochondria to produce an adequate quantity of ATP in response to the cell’s demand [89]. Mitochondria and the endoplasmic reticulum (ER) are the most important organelles involved in the control of metabolic flexibility, which is the ability to adapt metabolism to nutrient availability [90]. The chronic inflammatory state that characterizes obesity leads to an overproduction of reactive oxygen species (ROS), causing mitochondrial dysfunction [91]. Another reason is that an excessive amount of nutrients, overburdens the Krebs cycle and the respiratory chain [92]. CR and fasting intervene in this scenario via the activation of AMPK, which mediates the phosphorylation of several transcriptional co-activators of mitochondrial genes, such as peroxisomal proliferator-activated receptor-gammacoactivator1-alpha (PGC1α) and fork-head box O (FOXO) [93]. Furthermore, AMPK enhances the activity of sirtuins (SIRT), such as SIRT1 and SIRT3. SIRT1 plays a positive role in various cellular functions, including senescence, autophagy, lipid homeostasis and insulin sensitivity [94, 95]. Therefore AMPK and SIRT1 reciprocally activate in response to CR [96]. On the other hand, SIRT3 is positively involved in numerous mitochondrial functions, such as the protection from ROS and deacetylation of several proteins involved in fatty acid oxidation [97], in respiratory chain [98] and in cell death programs [99]. Mitochondria cooperate with ER in the maintenance of metabolic flexibility and energy metabolism [100]. ER membranes express inositol 1,4,5-trisphosphate receptor (IP3R), essential for the diffusion of Ca2 + from ER to mitochondria [101]. Alterations in this mechanism are involved in the development of metabolic disfunction such as insulin resistance [102] and metabolic dysfunction-associated steatotic liver disease (MASLD) [103]. During fasting state, cAMP can phosphorylate IP3R enhancing the interaction between ER and mitochondria [90]. Castro-Sepulveda and colleagues demonstrated that fasting-to-feeding transition reduces the interaction between ER and mitochondria and reduces mitochondrial cristae density in human peripheral blood mononuclear cells (PBMCs) [104].

### Cellular Senescence

Mitochondrial dysfunction is strictly connected with cellular senescence and can be both the cause and consequence of this condition. Senescence refers to the permanent state of cell cycles arrest, that occurs in proliferating cells, due to various kinds of cellular stresses. When senescent cells accumulate, the aging process starts, leading to functional decline and increase in morbidity and mortality [105]. Fasting can affect cellular senescence in several ways. Several biochemical pathways have been shown to promote longevity, among these, a central role is reserved for AMPK and mTOR pathways [106].

Interesting findings regarding activating transcription factor 4 (ATF4) have been discovered. ATF4, in fact, is a transcriptional factor with different target genes involved in cell survival, apoptosis, autophagy and protein synthesis, in response to various stress conditions [107]. It has been shown that ATF4 has a circadian behavior, with its levels rising with light and decreasing with darkness, as CLOCK:BMAL1 transactivates the ATF4 gene, while ATF4 controls *Per2* gene transcription [108]. Additionally, elevation in ATF4 levels in liver and fibroblasts seems to increase longevity and lifespan in mice [109]. Quiros and his colleagues found that ATF4 is the main factor activated in response to mitochondrial stress and mitonuclear stress [110]. On the other hand, in response to a fasting phase, ATF4 stimulates the transcription of *FGF21* gene [111]. FGF21 has multiple positive effects on metabolism, particularly on insulin sensitivity, body weight and glycemic control, and it is also associated with an increased lifespan in mice [112]. Taken together, these findings suggest that ATF4 pathway is another possible mechanism that could mediate the beneficial effects of TRE, but its role must be examined more in depth.

Additionally, fasting can decrease cell senescence by enhancing the activity of various members of the sirtuin family [113]. By decreasing SIRT4 levels, fasting rises the levels of α-ketoglutarate (αKG), an endogenous metabolite [114] capable of reducing the senescence-associated secretory phenotype (SASP) and inhibiting mTOR signaling pathway, while increasing AMPK activity [115]. In Drosophila*,* starvation induces SIRT4 and its deficiency correlates with decreased cellular longevity [116]. Conversely, CR and fasting reduce oxidative-stress and DNA damage by enhancing SIRT3 activity [117, 118]. Furthermore, in a study on patients with overweight or obesity, TRE appears to enhance SIRT1 expression [86].

Another essential coenzyme involved in cellular redox reactions is NAD. Nicotinamide phosphoribosyltransferase (NAMPT), also known as visfatin or pre-B cell colony enhancing factor (PBEF), is a controller of intracellular NAD + level [119]. NAMPT transcription is activated in a circadian manner by CLOCK and BMAL1 forming a complex with SIRT1 [120]. The regulation of its transcription leads to circadian oscillation of NAD + levels [121]. It has been shown that NAMPT-controlled NAD + levels can promote cell survival [122], starvation, in turn, increases both the levels of NAMPT and NAD + [123]. Interestingly, while NAMPT is expressed in all human tissue, it is highly expressed in adipose tissue and its circulating levels correlate with obesity and metabolic syndrome [124].

### NLRP3 Inflammasome and Chronic Low-Grade Inflammation

It is well-demonstrated that obesity is characterized by a chronic low-grade systemic inflammation [125]. Briefly, dysfunctional adipose tissue and consequent lipotoxicity lead to the development of a pro-inflammatory immune infiltration, resulting in the production of adipokines and cytokines, and ultimately establishing systemic inflammation [126]. Among various pathways, obesity-related inflammation is particularly influenced by the activation of the NOD-like receptor family pyrin domain containing 3 (NLRP3) inflammasome [127]. NLRP3 is a complex of the innate immune system that activates in response to various pathogen-associated molecular patterns (PAMPs) and/or danger-associated molecular patterns (DAMPs), leading to the production and activation of IL-18 and IL-1β and consequently amplifying systemic inflammation [128, 129]. The NLRP3/IL-1β pathway is also implicated in inducing insulin-resistance [130, 131]. An increasing body of evidence demonstrates that fasting reduces NLRP3-mediated inflammation via activation of SIRT3. For instance, a study showed that after 48 h of fasting, macrophages from wild-type mice produce lower quantities of IL-1β compared to those from SIRT3 knock-out mice. Additionally, SIRT3 reduces NLRP3 activity by inducing superoxide dismutase 2 (SOD2), thereby decreasing mitochondrial ROS levels [118]. Similar effects have been demonstrated by measuring IL-1β and IL-18 levels in human PBMCs during fasting and re-feeding: levels are lower during fasting and increase after re-feeding [132]. Moreover, it has been shown that after 24 h of administering a SIRT3 agonist to healthy volunteers, IL-1β and IL-18 production from PBMCs followed a similar trend as observed in the fasting state [132]. Based on previous studies demonstrating how NLRP3 inhibition ameliorates insulin-resistance [133, 134], Liang and colleagues demonstrated that fasting improves insulin sensitivity in mice and reverses insulin resistance in vitro, specifically in adipocytes used as a cellular model of insulin resistance. They also found that fasting inhibits the production of inflammatory markers (c-reactive protein, IL-1β, IL-18) induced by insulin resistance. This inhibition was counteracted by an NLRP3 agonist used to treat adipocytes in vitro, suggesting that the inhibition of NLRP3 induced by fasting may play a role in improving insulin sensitivity [135]. Furthermore, the inhibition of NLRP3 inflammasome by injecting a selective inhibitor into the peritoneum of mice after re-feeding reduced the production of IL-1β in hepatocytes and ameliorated fasting-induced hepatic lipid deposition [136]. Although an evaluation on NLRP3 activity was not performed, another study showed decreased levels of IL-1β and reduced inflammatory response in hepatocyte of mice treated with every-other day feeding regimen [137]. Inhibiting NLRP3 and other members of the NLRP3 family (such as NLRP1) through intermittent fasting also reduces cells death following chronic and acute hypoperfusion [138, 139].

### Metabolic Signaling and Nutrients’ Sensing Pathways

TRE can control several metabolic signaling pathways in different organs, such as adipose tissue, liver and muscle [14, 140, 141]. In adipose tissue, prolonged fasting influences the expression of genes such as *GLUT4*, *IRS2* and *AKT*. Specifically, it enhances the transcription of perilipin 2 and ATG, involved in lipolysis, while reducing perilipin 1. It reduces the transcription of adiponectin, adiponectin receptor, insulin-like growth factor-1 (IGF-1), resistin and leptin, although contrasting data has been collected on factors related to hemostasis, angiogenesis and immunity [142]. Conversely, in adults affected by overweight and obesity, late isocaloric eating enhanced the expression of genes involved in lipogenesis (*GPAM, ACLY, AACS,* and *CERK*), while reducing those involved in lipid breakdown (*PLD6, DECR1,* and *ASAH1*), as well as lipolysis genes such as *ABDH5*, which is inhibited by perilipin 1 [143].

Regarding insulin signaling, fluctuations in insulin levels during fasting and feeding cycles interact with the clock machinery. For instance, TRE can control *per2* expression by reducing IGF-1 and insulin production during fasting [144]. Goldbraikh et al., demonstrated that during fasting, the ubiquitin‐specific protease 1 (USP1) de-ubiquitination activity is reduced, with a consequent increase in Akt ubiquitination, PI3K‐Akt‐FoxO signaling, and glucose uptake [145]. Also in humans, early-TRE may improve insulin-sensitivity by increasing the expression of AKT2, a downstream member of the insulin signaling pathway [86]. Fasting seems also to enhance the phosphorylation of insulin receptor substrates 1 and 2 (IRS1 and IRS2) with a consequent increase in the association with PI3-kinase in liver and muscle [146]. Glucose starvation increases vacuolar H + -ATPase (V-ATPase) assemblage [147], a proton pumps that controls glycolysis, insulin secretion, glucose transporter and that takes part in various pathophysiological mechanisms leading to diabetes [148]. SREBP-1c, a member of the sterol regulatory element binding proteins (SREBP) family, is a downstream effector of the insulin signaling, involved in fatty acids synthesis and insulin induced metabolism [149]. In diabetics rats’ liver, intermittent fasting seems to reduce SREBP1-c and increase PPAR-γ levels [150], in mice liver also, SREBP1-c levels were reduced after starvation [151]. A study on humans showed that after 48 h of starvation, in skeletal muscle the expression of SREBP1-c is decreased, whereas IRS1 and IRS2 levels do not change [152]. However, in adipose tissue of patients with obesity obese and type 2 diabetes, SREBP-1c mRNA expression was found to be decreased in comparison with lean subjects [149]. In the liver, the fasting-feeding cycle also drives rhythmic expression of other molecular participants in metabolic pathways. In mice fed with ad libitum high-fat diet, hepatic levels of pCREB are constitutively elevated. CREB controls lipolysis, lipogenesis and particularly gluconeogenesis in liver [153]. In mice fed with a normal chow or high-fat diet under a time-restricted feeding regimen, several changes were observed: 1) a restoration of daytime peaks in pCREB, 2) increased levels of the transcriptional repressor Rev-erb-α, leading to the repression of fatty acid synthase and reduction in levels of several long-chain free fatty acids, and 3) increased expression of Per2, which inhibits PPARγ and subsequently attenuates the transcription of lipogenic genes [29, 45]. Furthermore, CREB is an activator also of the coactivator 1α (PGC1α). The axis CREB/PGC1α, according to Besse-Patin et al., activates genes that trigger lipid catabolism and gluconeogenesis during fasting [154]. As shown also in chapter 2, these findings are supported by previous research demonstrating how both the temporal pattern of food intake and circadian clock rhythmically regulate the transcription of numerous hepatic genes, including those involved in metabolic pathways such as *CREB*, *AKT*, and *AMPK* [40, 41].

## Effects of TRE on Metabolism and Appetite Control Players

Restoring the tissues’ circadian expression of genes involved in metabolic pathways could be the key for protecting against metabolic disorder caused by excessive food intake and consequent obesity. Obesity is characterized by mitochondrial dysfunctions and systemic low-grade inflammation, both important drivers for the development of its comorbidities and complications. A dietary regimen that blunts this inflammatory status and promotes mitochondrial activity could be the initial step in a comprehensive therapeutic program. However, one of the most important factors for the adherence to a dietary protocol is appetite control, while the adherence to a dietary prescription is the best predictor of weight loss, over the short and long term [155]. Therefore, we decided to explore the effects of TRE on hormones involved in appetite control [156, 157], summarized in Table 1.

**Table 1:** Hormones and neurotransmitters involved in appetite regulation

Tissue / organ	Hormone or neurotransmitter	Effect on appetite	TRE influence
Hypothalamus	NPY AgRP Orexin MCH POMC TRH CRH CART	Orexigenic Orexigenic Orexigenic Orexigenic Anorexigenic Anorexigenic Anorexigenic Anorexigenic	↑ None None Not known Conflicting evidence Not known ↓ None
Gut	Ghrelin CCK PYY GLP-1	Orexigenic Anorexigenic Anorexigenic Anorexigenic	↓/ = ↓ Conflicting evidence ↓/ =
Pancreas	PP Amylin Insulin	Anorexigenic Anorexigenic Anorexigenic	↑ None Conflicting evidence
Adipose tissue	Leptin Adiponectin	Anorexigenic -	↓ ↑
Gut microbiota	SCFAs	Anorexigenic	↑

*NPY*: neuropeptide *Y*; *AgRP*: agouti-related peptide; *MCH*: melanin-concentrating hormone; *POMC*: pro-opiomelanocortin; *TRH*: thyroid releasing hormone; *CRH*: corticotropin releasing hormone; *CCK*: cholecystokinin; *PYY*: peptide YY; *GLP-1*: glucagon-like peptide-1; *PP*: pancreatic polypeptide

### Hypothalamic Hormones

Appetite is regulated by a complex integration of several neuronal and peripheral signaling [158]. Briefly, the hypothalamus arcuate nucleus (ARC) plays a pivotal role, expressing hormones’ receptors such as leptin, ghrelin and insulin and thus acting as an energy sensor [159]. ARC includes neurons releasing neuropeptide Y (NPY) and agouti-related peptide (AgRP) that stimulate food intake, and pro-opiomelanocortin (POMC) cells expressing α-melanocyte-stimulating hormone (α-MSH) and cocaine- and amphetamine-regulated transcript (CART) that inhibit food intake. NPY/AgRP and α-MSH/CART receptors are expressed on several hypothalamic nuclei, such as paraventricular nucleus (PVN), dorsomedial hypothalamus (DMH), lateral hypothalamic nucleus (LHA) and ARC itself for feedback regulation. PVN releases thyroid releasing hormone (TRH), corticotropin releasing hormone (CRH) and oxytocin which show anorexigenic effects. LHA releases melanin-concentrating hormone [66] and orexin A and B, which act as orexigenic hormones [160].

In fasting protocols, NPY expression has been extensively studied [157]. Intermittent fasting increases the expression of NPY mRNA in ARC [161–164], thus suggesting a counterregulatory process to short-term weight loss induced by calorie restriction. However, in another study NPY mRNA expression was not influenced by intermittent fasting [165]. Yoshihara et al. demonstrated that in rats under restricted feeding conditions NPY levels showed a pre-feeding peak, suggesting that NPY is also regulated by the fasting-feeding circadian rhythm [166].

On the other hand, conflicting evidence exists regarding POMC concentrations: after intermittent fasting, they have been found to be either increased [163] or unchanged [164]. Mice knockout for melanocortin 4 receptor (MC4R, the α-MSH receptor), do not show a significant weight loss when compared to wild type mice fed according to a dark restricted feeding protocol (in other words, fed only in the active phase). This evidence suggests that melanocortin system may play a role in weight loss during TRF [167].

Orexin, AgRP and CART expression are less studied, but it seems they are not influenced by intermittent fasting [164, 168].

### Gut Hormones

Ghrelin is mostly secreted by X/A cells in gastric fundus. Its levels reach their maximum during fasting and minimum just after the meal [169]. Ghrelin increases gastric motility and secretion in advance of food ingestion and it stimulates arcuate nucleus receptors in the hypothalamus, inducing the production of NPY and AgRP and therefore stimulating appetite [170].

Cholecystokinin (CCK) is secreted by duodenal and jejunal I cells after meals, especially high-fat ones, when it stimulates the secretion of bile and pancreatic enzymes and, binding its receptors on vagus nerve, suppresses hunger [171].

Peptide YY (PYY) is secreted by ileum and colon L cells proportionally to calorie intake after meals and exerts its anorexigenic function at the level of the arcuate nucleus, inhibiting NPY and PYY secretion [172].

Glucagon like peptide-1 (GLP-1) is produced by intestinal L cells in response to glucose presence in the luminal intestinal space. It stimulates insulin secretion, inhibits glucagon production, slows gastric emptying and reaches hypothalamus to promote satiety and reduce food intake [173].

Subjects affected by obesity have low levels of ghrelin during fasting and show an impaired suppression of ghrelin secretion after meal, as an adaptive response to chronic positive energy balance [170]. Furthermore, they show lower post-prandial levels of GLP-1 and PYY [172], suggesting that satiety is not reached, and that the next meal will be desired earlier [173]. During dietary intervention, PYY and CCK levels were reduced, while ghrelin levels were increased, with a consequent reduction of the feeling of fullness [171]. This is one of the most powerful compensatory responses that counteract weight loss [174].

Even if TRE seems to improve satiety and appetite control, evidence about how it influences gut hormones production is less consistent [156, 175]. In animal models of restricted feeding protocols, when compared to ad libitum protocols or high fat diet, ghrelin levels have been found both lower [176] and higher [167, 177]. In humans, earlyTRE lowered the fasting levels of ghrelin [178, 179]. These results are in line with a previous study, showing that if caloric intake is higher at breakfast than at dinner, the levels of ghrelin, insulin and fasting glucose decrease when compared to an opposite distribution of caloric intake [180]. A study on Ramadan period showed a decrease in ghrelin levels during daytime [181], while in other studies there were no effects on ghrelin concentrations [175, 182–185]. In contrast, only one study showed that a period of alternate- day fasting led to an increase in ghrelin [186].

Evidence about PYY and GLP-1 levels is controversial. In humans studies, TRE was found to decrease [182, 187], increase [178] or not affecting at all [179, 188] PYY concentrations.

According to some evidence, TRE reduces fasting GLP-1, while it has no effect on postprandial GLP-1 [178, 179], but in other studies GLP-1 fasting levels were not affected by TRE [187, 188].

Finally, only one study explored CCK concentrations in humans and showed decreased levels after Ramadan period [182].

### Pancreatic Hormones

Pancreatic polypeptide (PP) is mostly secreted by pancreatic islets of Langerhans PP cells and to a lesser extent by intestinal cells after meals. Its functions are to slow down stomach emptying, intestinal motility and pancreatic enzymes secretion. Moreover, via the vagus nerve, it inhibits hunger and stimulates satiety [189].

Amylin is secreted by pancreatic $\upbeta -$ cells (together with insulin) in response to nutrients intake. It acts as anorexigenic hormone, suppressing glucagon secretion and stimulating satiety [190].

Insulin is produced by β-cells in the pancreatic islets of Langerhans. After meals, glucose level rises, and β-cells release insulin. Binding its receptors on skeletal muscle, adipose tissue and liver, it allows glucose to enter the cells in order to produce energy [191]. Insulin reaches also some brain receptors, crossing the blood-barrier via transporters, in order to decrease food intake, with anorexigenic effect [192]. When glucose levels arise because of excessive food intake, β-cells improve their function to increase glucose uptake, resulting in hyperinsulinemia. Furthermore, the presence of pro-inflammatory cytokines, above all TNF alpha, IL-6 and IL-1, causes insulin receptor downregulation, with decreased signaling glucose uptake. These modifications lead to the establishment of insulin resistance, where insulin hematic concentration is increased but with no effect on its receptor [193].

Most of the studies concentrated on insulin secretion, while amylin and PP are poorly studied in TRE or under fasting conditions [156]. Only one study investigated PP levels after five days of absolute fasting in healthy humans, and found that PP serum concentrations increased progressively [194]. As regards amylin, in a study by Hutchinson et al. TRF had no effect on fasting or postprandial amylin levels [179].

Several evidence show that TRE do not influence fasting and postprandial insulin levels [156, 179, 188, 195], while other studies suggest TRE effectiveness in reducing insulin levels and ameliorating insulin-resistance [196]. Although insulin signaling has been extensively studied in the context of TRE, studies have proposed different definitions of TRE in different populations, making it difficult to draw firm conclusions [156]. For example, Barnosky et al. demonstrated that there is no difference in reducing fasting insulin levels between CR and fasting protocols [197]. In contrast, TRE reduced insulin levels, in women with polycystic ovarian syndrome [198], in subjects with type 2 diabetes [199], in men with prediabetes [187] and in healthy individuals [200]. Clinical outcomes of TRE on insulin sensitivity in the context of obesity are discussed more in detail in paragraph 5.

### Adipose Tissue and Adipokines

Far from being a simple depot, adipose tissue acts as a true endocrine organ capable of producing adipokines, chemokines and other molecules that interact with other tissues [201].^.^ Alterations in the quantity, quality and distributions of adipose tissue can cause dysfunctions in its complex regulation [202].

Leptin and adiponectin are primarily secreted by white adipose tissue, but also by other tissues such as bone marrow and mammary epithelial cells. Leptin induces satiety and decreases food assumption. Adiponectin promotes fatty acid oxidation and decreases fat mass accumulation and gluconeogenesis. Its levels are inversely proportional to fat mass [201]. In obesity, the levels of leptin increase proportionally to fat mass, while its receptors’ sensibility decreases resulting in a loss of anorexigenic action, in a process called leptin-resistance [203]. Adipokines release is strictly connected with insulin sensitivity, therefore modifications in leptin and adiponectin levels may be due to an improvement in insulin sensitivity, in fact fasting insulin reduction and HOMA-IR improvement has been recorded after fasting protocols [204]. This interaction is linked especially with adiponectin, that promotes muscle fatty acids oxidation and inhibits liver gluconeogenesis through the activation of AMPK to increase GLUT1 and GLUT4 receptors expression, to improve glucose uptake, activating glycolysis and reducing liver gluconeogenesis [205].

In general, several studies reported that TRE and intermittent fasting increase adiponectin levels, while decrease leptin concentrations [156, 196]. When compared to caloric restriction, TRE showed greater reduction in leptin levels, an increase in adiponectin levels and a decrease in leptin/adiponectin ratio in both overweight women [174] and normal weight men [204]. Another study showed leptin reduction both in caloric restriction and in fasting protocols, independent of weight loss [206]. However, other data show that leptin levels do not change if TRE is not associated with caloric restriction [187, 188].

### Gut Microbiota

Increasing burden of evidence shows that gut microbiota has a therapeutic potential in many pathophysiological mechanisms.

Firmicutes and Bacteroidetes represent about 90% of all bacteria present in gut [207]. Obesity is associated with dysbiosis, particularly with a reduced diversity in bacterial components, and an increased presence of Firmicutes instead of Bacteroidetes [208]. This dysbiosis influences the secretion of hormones such as PYY, CCK and leptin, resulting in an alteration of central satiety mechanisms [209, 210].

Intestinal bacteria produce short-chain fatty acids (SCFAs) via fiber fermentation. SCFAs can control satiety by the activation of G protein-coupled receptor 41 (GPR41) and GPR43, thus inducing the production of GLP-1, PYY, and leptin [211, 212]. Bacteroidetes are positively associated with higher concentrations of SCFAs, while Firmicutes show an inverse relationship [213].

TRE in humans determines an increase in richness and diversity of gut microbiota [64, 214, 215]. Moreover, when TRE was performed during the early hours of the day, the effect was even more marked [215].

Intermittent fasting increases the production of SCFAs [214, 216–218] also by inducing an increase in Prevotellaceae, which can in turn enhance the production of SCFAs [219].

Furthermore, intermittent fasting enhances the presence of Lactobacillus spp. and Akkermansia, both associated with positive effects on metabolic regulation [212, 214, 215]. Indole, a catabolite produced by Bacteroides, Lactobacillus, and Bifidobacterium, can enhance GLP-1 release, thus improving appetite regulation [212, 220].

However, in other studies, fasting protocols did not influence SCFAs production, both in humans and in mice [213, 221].

### Appetite Control and Circadian Rhythm

The disruption of circadian rhythm, with an increase in evening activities and the delay in the last meal of the day, determines a dysregulation in many physiological processes such as basal metabolism, hormone secretion, body temperature regulation and sleep/wake cycle, that altogether determines an increase in cardiovascular risk. Also appetite control follows the circadian rhythm, and the restoring of the inner biological clock could be helped by the resynchronization of food intake with the physiological hormonal secretion [222].

Melatonin secretion is fundamental in guiding daily rhythms [223]. In the first hours of daylight, melatonin levels decrease with a reduction in inhibiting cortisol production. Therefore, cortisol levels reaches its peak at 8 am circa, together with ghrelin, in order to stimulate appetite for the increase in energetic demands induced by morning activity [224]. Ghrelin has a second and third peak also round 1 pm and 6 pm and its levels decrease after meal [223]. Adiponectin reaches its peak at 11 am, promoting glucose uptake and avoiding fat accumulation, while its secretion ends at 8 pm [223]. Insulin reaches peak at 5 pm stimulating fatty acids synthesis and inhibiting gluconeogenesis and fatty acid oxidation, leading to fat accumulation [225]. PYY land PP levels counteract food intake during daytime since its levels are higher during the day than during the night [169, 226]. Leptin levels start rising in the first afternoon and reach a peak in the first few hours of the habitual sleep timing and decrease across the remainder of the sleep episode in order to suppress hunger during the inactive phase [169, 223]. Following this circadian oscillation of hormone production, it can be supposed that the last meal should not be later than 6 PM [169].

Therefore, eating earlier in the day should be preferred with respect to evening meals [227]. Different studies showed that eating at nighttime was associated with greater caloric intake, more frequent meals and at more irregular times [228]. As we have shown, fasting protocols proposing meals early in the day, compared to evening or night eating, improve diet quality, reduce caloric intake [229], reduce snacking [230], regulate eating times and also reduce appetite, leading to greater weight loss [231–234].

Dashti et al. [235] examined a large cohort of subjects affected by obesity during weight loss intervention and divided them in early and late eaters, showing that weight loss is lower in late eaters, which also exhibited a dysregulation in the secretion of the hormones involved in appetite control. In the same way, Vujović et al. [143] hypothesized that late eating could promote positive energy balance increasing the drive for energy intake in relation to increased ghrelin and decreased leptin levels.

## Clinical Outcomes in the Context of Obesity

We have seen how TRE produces direct effects on cellular energy metabolism and hormonal regulation of appetite. It is therefore interesting to look at the results of clinical studies on TRE conducted in the context of obesity, focusing on weight loss and cardiovascular risk factors such as glycemic control, lipid profile and blood pressure.

Clinical studies on TRE in humans are very recent. In literature, we can only find them starting from the late 2010s, with a noteworthy increase in available data from 2022. In Supplementary Table 1, we summarized the main clinical trials on adult subjects affected by obesity, indicating the type of intervention applied, the study population and the most relevant findings.

It is important to note that these studies encompass various types of interventions under the “umbrella term” of TRE. TRE refers to dietary interventions that require eating within a specific timeframe, regardless of its duration [14]. Most studies [188, 236–247] applied the common "8-h" TRE, which involves an 8-h eating window followed by a 16-h fasting period, and only one study evaluated narrower 4- and 6-h eating windows [248]. Some studies defined also a more precise eating window, that for example had to start in 3 h from waking up (early-TRE) [249]. Usually during the fasting period only non-caloric liquids (water, tea, coffee) are allowed, while guidelines for the eating window are less stringent. A minority of the studies also incorporated calorie restriction in addition to TRE [241, 249–252], only one study requested a “isocaloric” TRE (further discussed in the next paragraph) [253] while the others simply did not request calorie counting. As a result, these studies presented different types of interventions, varying in duration (ranging from 5 days [188]) to 12 months [240, 241, 245]) and evaluated outcomes (for example, not all the studies registered body weight or blood pressure variation). This heterogeneity creates challenges in drawing firm conclusions on clinical effects of TRE.

### Weight Loss

Regarding weight loss, trials consistently show that TRE can induce significant weight loss compared to baseline, even in short periods such as three weeks [250]. However, most randomized controlled trials comparing TRE with caloric restriction have not demonstrated significant differences in weight loss between the two regimens [236, 240, 243, 244, 254]. These results indicate not a lack of effectiveness of TRE in inducing weight loss, but rather an effect comparable to that of more common CR regimes. Only two studies showed a significantly higher impact of TRE on weight loss compared to CR [239, 246]. Interestingly, in the randomized controlled trial by Kotarsky and colleagues both TRE and CR groups underwent concomitant exercise training, suggesting that the use of TRE and concurrent exercise training could reduce fat mass and increase lean mass in a more favorable way than CR [239]. In two other studies, participants who underwent exercise training (such as high intensity interval training or functional training) while applying TRE achieved higher weight loss compared to those who only did TRE [247, 255]. These results reinforce the indication that a comprehensive lifestyle intervention should always include an increase in physical activity, but they also suggest that the best weight loss results are achieved by creating an energy balance that is as negative as possible. The weight loss effect of TRE may be primarily due to reduced caloric intake: indeed, a study found that patients practicing TRE reduced their energy intake by ∼550 kcal/day compared to controls, without calorie counting [200]. Moreover, in a randomized controlled trial testing a 10-h isocaloric TRE (that means, a protocol in which the participants would continue to consume their usual daily calories but within a restricted time window) for 12 weeks, there was no difference in weight loss between TRE and controls [253]. Therefore, reducing calorie intake is key for weight loss.

Restricting the eating window decreases the likelihood of consuming excess calories and can yield better weight loss results, as shown in a study that compared 10-h TRE to 12-h TRE [256]: in 8 weeks both groups achieved a satisfactory weight loss compared to baseline, but there was a significant difference in favor of the 10-h TRE group. This suggests that eating windows of less than 8 h may achieve even better results, but in the study of Cinfuegos and colleagues 4-h and 6-h TRE achieved comparable results in the same 8 weeks period [200], highlighting a need for studies directly comparing TRE regimens with eating window difference higher than 2 h.

A fundamental element in dietary interventions is adherence to the diet itself. Most studies agree in defining TRE as "feasible" [257, 258]. The simplicity of not needing to count calories can be liberating for some patients, who may find it easier to follow a simple time-based guideline rather than a specific and oppressive diet plan [259]. Adherence is directly correlated with weight loss, as demonstrated by a study in which participants who adhered to TRE for more than 5 days a week achieved better results than those who did not [238]. Nowadays, technology offers new tools to incentivize adherence to therapeutic prescriptions [260]: in a study by Prasad and colleagues, participants received push notifications on their smartphones that alerted them to the opening and closing of the TRE window, achieving better weight loss results compared to controls [261]. Further studies that include these new technologies are necessary to understand if the approach is cost-effective.

In summary, TRE can be a valid alternative to CR in the treatment of obesity, as it is a regimen that in adherent patients can lead to clinically meaningful weight loss reducing overall calorie intake, but more studies are needed to define the best TRE protocol.

### Cardiovascular Risk Factors

A comprehensive treatment for obesity should address not only weight loss, but also to the control of important cardiovascular risk factors such as insulin-resistance, atherogenic lipid profile and high blood pressure.

We have seen that from the “cellular” point of view TRE has the potential for ameliorating glucose metabolism. In a randomized controlled crossover trial, 11 men with obesity followed a 8-h TRE protocol and at the 5th day underwent a 24-h laboratory assessment: area under the curve for venous glucose tended to be lower for TRE compared to controls [188], suggesting a potential role for TRE in restoring insulin-sensitivity. Other non-randomized studies showed a slight reduction in fasting glucose or Hb1Ac [262, 263], however most of the randomized controlled studies in patients with obesity but without type 2 diabetes agree that TRE do not have a significant impact on glycemic control or insulin sensitivity [188, 236, 239–243, 245, 249–253]. Differently, TRE induced a reduction in Hb1Ac when associated with physical activity (high intensity interval training) [255], but in this case the effect could be mediated by the weight loss achieved.

Evidence on TRE in obesity complicated by type 2 diabetes is less consistent. When compared to mediterranean diet, a TRE protocol specifically designed to remodulate the daily uptake of carbohydrates failed to achieve a better glycemic control after 12 weeks of treatment [264], while a 8-h TRE regimen induced a significant reduction in Hb1Ac in 6 months, comparable to that of patients that underwent a CR intervention [246]. These results suggest that a potential confounder can be also the duration of the follow-up: probably shorter interventions have less potential to determine an impact on Hb1Ac, even if insulin-sensitivity is temporarily restored. Furthermore, even if the 8-h TRE is the most studied protocol, the eating windows can vary: according to our circadian rhythm, the most effective window is likely from early morning to early afternoon, but most of the studies used the noon-to-dinner window [15].

In terms of duration of the eating window, no differences in fasting glucose were evidenced when 4-h and 6-h TRE regimens were compared [248], as well as 10-h and 12-h TRE [256]. Also in this case, there is a need for studies comparing TRE regimens with longer eating window difference.

Finally, in the context of type 2 diabetes it is also important to pay attention to possible side effects of prolonged fasting in patients using hypoglycemic drugs [199]. For insulin-dependent patients, it is reasonable to assume that insulin therapy may need to be adjusted according to the prescribed eating schedule.

As regards lipid profile, only a single-arm trial showed a reduction in LDL cholesterol with 10-h TRE in 12 weeks [262], while all randomized controlled studies agree that TRE do not induce a significant variation in LDL cholesterol [236, 237, 240, 241, 245, 248, 249, 252, 255], regardless of the duration, the type of intervention and the weight loss achieved. However, if associated with organized physical activity, TRE seems to reduce total cholesterol and triglycerides levels [247, 265] and increase HDL cholesterol [255], thus suggesting an important role for exercise rather than fasting.

In the same way, evidence from all randomized controlled trials show that TRE does not present additive beneficial effects on systolic and diastolic blood pressure when compared to other weight loss interventions in the context of obesity [236, 237, 240, 241, 245, 250–252, 255]. However, it is important to underline that also in this case TRE represents a valid alternative to CR: in the study by Liu and colleagues (that is the longest trial on TRE in obesity, lasting 12 months) 8-h TRE determined a mean 8.0 kg weight loss and a consistent reduction of 8.1 mmHg in systolic blood pressure and of 5.1 mmHg in diastolic blood pressure, slightly more (but the difference is not statistically significant) than CR [240].

### Metabolic-Dysfunction Associated Steatotic Liver Disease

An emerging and interesting field of study is metabolic-dysfunction associated steatotic liver disease (MASLD), a very common and early complication in patients affected by obesity [266]. When compared to mediterranean diet, TRE seems to reduce both intrahepatic stiffness and hepatic steatosis assessed via controlled attenuation parameter (CAP) [267]. Early-TRE and late-TRE equally determined a significant reduction of intrahepatic fat and improvements in liver function [268]. However, another study measured intrahepatic triglycerides content via magnetic resonance imaging (MRI) after a period of 6 months of TRE and then after 6 months of additional follow up, showing no differences between TRE and control group [244]. In summary, TRE seems to be a dietary strategy to take into consideration also for patients with MASLD to reduce hepatic steatosis and ameliorate liver function [269], but further studies are needed.

## Conclusions and Future Perspectives

Circadian misalignment is a significant risk factor for the development of metabolic diseases such as obesity, metabolic syndrome and type 2 diabetes. TRE, a dietary approach that limits food consumption to a specific window of the day, has emerged as a novel dietary strategy to face obesity and its associated metabolic complications. Fasting/feeding cycle acts as an important *zeitgeber* for peripheral clocks and restricting eating to daylight time helps restore the circadian expression of several molecular targets. TRE positively affects metabolic pathways involved in mitochondrial functions, autophagy, senescence, inflammation, insulin signaling, lipid productions and storage.

However, clinical trials examining if these effects can determine clinical benefits for people suffering from obesity have shown less consistent evidence. TRE can lead to significant weight loss compared to baseline, mainly due to reduced calorie intake. Its efficacy correlates with adherence and the duration of the fasting phase, and, in fact, the narrower the eating window, the better the result. However, clinical trials failed to show a net benefit of TRE compared to other weight loss strategies. Regarding cardiometabolic risk factors, TRE does not appear to provide an advantage in terms of fasting glucose, lipid profile or blood pressure.

However, TRE seems to be safe and at least non-inferior to caloric restriction in terms of clinical outcomes, thus emerging as an alternative option in patients who have difficulty and poor adherence in following other dietary strategies.

The vast heterogeneity in clinical trials in terms of population, intervention and follow-up does not allow us to draw firm conclusions, thus more studies are needed to define TRE potential benefits in health and in which patients can be more effective.

## Supplementary Information

Below is the link to the electronic supplementary material.

Supplementary file1 (DOCX 30.9 KB)

## Data Availability

No datasets were generated or analysed during the current study.

## References

[1] Perdomo CM, Cohen RV, Sumithran P, Clément K, Frühbeck G. Contemporary medical, device, and surgical therapies for obesity in adults. Lancet. 2023;401(10382):1116–30. 36774932 10.1016/S0140-6736(22)02403-5

[2] Jensen MD, Ryan DH, Apovian CM, Ard JD, Comuzzie AG, Donato KA, et al. 2013 AHA/ACC/TOS guideline for the management of overweight and obesity in adults: a report of the American College of Cardiology/American Heart Association Task Force on Practice Guidelines and The Obesity Society. J Am Coll Cardiol. 2014;63(25 Pt B):2985–3023. 24239920 10.1016/j.jacc.2013.11.004

[3] Leibel RL, Rosenbaum M, Hirsch J. Changes in energy expenditure resulting from altered body weight. N Engl J Med. 1995;332(10):621–8. 7632212 10.1056/NEJM199503093321001

[4] Schwartz A, Kuk JL, Lamothe G, Doucet E. Greater than predicted decrease in resting energy expenditure and weight loss: results from a systematic review. Obesity (Silver Spring). 2012;20(11):2307–10. 22327054 10.1038/oby.2012.34

[5] Sainsbury A, Zhang L. Role of the hypothalamus in the neuroendocrine regulation of body weight and composition during energy deficit. Obes Rev. 2012;13(3):234–57. 22070225 10.1111/j.1467-789X.2011.00948.x

[6] Sumithran P, Prendergast LA, Delbridge E, Purcell K, Shulkes A, Kriketos A, et al. Long-term persistence of hormonal adaptations to weight loss. N Engl J Med. 2011;365(17):1597–604. 22029981 10.1056/NEJMoa1105816

[7] Melby CL, Paris HL, Foright RM, Peth J. Attenuating the Biologic Drive for Weight Regain Following Weight Loss: Must What Goes Down Always Go Back Up? Nutrients. 2017;9(5):468. 28481261 10.3390/nu9050468 PMC5452198

[8] Maclean PS, Bergouignan A, Cornier MA, Jackman MR. Biology’s response to dieting: the impetus for weight regain. Am J Physiol Regul Integr Comp Physiol. 2011;301(3):R581-600. 21677272 10.1152/ajpregu.00755.2010 PMC3174765

[9] Dulloo AG, Jacquet J, Montani JP, Schutz Y. How dieting makes the lean fatter: from a perspective of body composition autoregulation through adipostats and proteinstats awaiting discovery. Obes Rev. 2015;16(Suppl 1):25–35. 25614201 10.1111/obr.12253

[10] Sumithran P, Proietto J. The defence of body weight: a physiological basis for weight regain after weight loss. Clin Sci (Lond). 2013;124(4):231–41. 23126426 10.1042/CS20120223

[11] van Baak MA, Mariman ECM. Dietary Strategies for Weight Loss Maintenance. Nutrients. 2019;11(8):1916. 31443231 10.3390/nu11081916 PMC6722715

[12] Moon S, Kang J, Kim SH, Chung HS, Kim YJ, Yu JM, et al. Beneficial Effects of Time-Restricted Eating on Metabolic Diseases: A Systemic Review and Meta-Analysis. Nutrients. 2020;12(5):1267. 32365676 10.3390/nu12051267 PMC7284632

[13] Paoli A, Tinsley G, Bianco A, Moro T. The Influence of Meal Frequency and Timing on Health in Humans: The Role of Fasting. Nutrients. 2019;11(4):719. 30925707 10.3390/nu11040719 PMC6520689

[14] Tang D, Tang Q, Huang W, Zhang Y, Tian Y, Fu X. Fasting: From Physiology to Pathology. Adv Sci (Weinh). 2023;10(9):e2204487. 36737846 10.1002/advs.202204487 PMC10037992

[15] Petersen MC, Gallop MR, Flores Ramos S, Zarrinpar A, Broussard JL, Chondronikola M, et al. Complex physiology and clinical implications of time-restricted eating. Physiol Rev. 2022;102(4):1991–2034. 35834774 10.1152/physrev.00006.2022 PMC9423781

[16] Guglielmi V, Bettini S, Sbraccia P, Busetto L, Pellegrini M, Yumuk V, et al. Beyond Weight Loss: Added Benefits Could Guide the Choice of Anti-Obesity Medications. Curr Obes Rep. 2023;12(2):127–46. 37209215 10.1007/s13679-023-00502-7 PMC10250472

[17] Panda S, Hogenesch JB, Kay SA. Circadian rhythms from flies to human. Nature. 2002;417(6886):329–35. 12015613 10.1038/417329a

[18] Longo VD, Panda S. Fasting, Circadian Rhythms, and Time-Restricted Feeding in Healthy Lifespan. Cell Metab. 2016;23(6):1048–59. 27304506 10.1016/j.cmet.2016.06.001 PMC5388543

[19] Challet E. The circadian regulation of food intake. Nat Rev Endocrinol. 2019;15(7):393–405. 31073218 10.1038/s41574-019-0210-x

[20] Gaddameedhi S, Selby CP, Kaufmann WK, Smart RC, Sancar A. Control of skin cancer by the circadian rhythm. Proc Natl Acad Sci U S A. 2011;108(46):18790–5. 22025708 10.1073/pnas.1115249108 PMC3219110

[21] Manella G, Sabath E, Aviram R, Dandavate V, Ezagouri S, Golik M, et al. The liver-clock coordinates rhythmicity of peripheral tissues in response to feeding. Nat Metab. 2021;3(6):829–42. 34059820 10.1038/s42255-021-00395-7 PMC7611072

[22] Leng Y, Musiek ES, Hu K, Cappuccio FP, Yaffe K. Association between circadian rhythms and neurodegenerative diseases. Lancet Neurol. 2019;18(3):307–18. 30784558 10.1016/S1474-4422(18)30461-7 PMC6426656

[23] Mattis J, Sehgal A. Circadian Rhythms, Sleep, and Disorders of Aging. Trends Endocrinol Metab. 2016;27(4):192–203. 26947521 10.1016/j.tem.2016.02.003 PMC4808513

[24] Guo J, Huang X, Dou L, Yan M, Shen T, Tang W, et al. Aging and aging-related diseases: from molecular mechanisms to interventions and treatments. Signal Transduct Target Ther. 2022;7(1):391. 36522308 10.1038/s41392-022-01251-0 PMC9755275

[25] Bunger MK, Wilsbacher LD, Moran SM, Clendenin C, Radcliffe LA, Hogenesch JB, et al. Mop3 is an essential component of the master circadian pacemaker in mammals. Cell. 2000;103(7):1009–17. 11163178 10.1016/s0092-8674(00)00205-1 PMC3779439

[26] Dunlap JC. Molecular bases for circadian clocks. Cell. 1999;96(2):271–90. 9988221 10.1016/s0092-8674(00)80566-8

[27] Vitaterna MH, King DP, Chang AM, Kornhauser JM, Lowrey PL, McDonald JD, et al. Mutagenesis and mapping of a mouse gene, Clock, essential for circadian behavior. Science. 1994;264(5159):719–25. 8171325 10.1126/science.8171325 PMC3839659

[28] Takahashi JS. Transcriptional architecture of the mammalian circadian clock. Nat Rev Genet. 2017;18(3):164–79. 27990019 10.1038/nrg.2016.150 PMC5501165

[29] Cho H, Zhao X, Hatori M, Yu RT, Barish GD, Lam MT, et al. Regulation of circadian behaviour and metabolism by REV-ERB-α and REV-ERB-β. Nature. 2012;485(7396):123–7. 22460952 10.1038/nature11048 PMC3367514

[30] Mukherji A, Kobiita A, Chambon P. Shifting the feeding of mice to the rest phase creates metabolic alterations, which, on their own, shift the peripheral circadian clocks by 12 hours. Proc Natl Acad Sci U S A. 2015;112(48):E6683–90. 26627259 10.1073/pnas.1519735112 PMC4672831

[31] Stokkan KA, Yamazaki S, Tei H, Sakaki Y, Menaker M. Entrainment of the circadian clock in the liver by feeding. Science. 2001;291(5503):490–3. 11161204 10.1126/science.291.5503.490

[32] Patterson RE, Sears DD. Metabolic Effects of Intermittent Fasting. Annu Rev Nutr. 2017;37:371–93. 28715993 10.1146/annurev-nutr-071816-064634 PMC13170603

[33] Brown PJ, Konner M. An anthropological perspective on obesity. Ann N Y Acad Sci. 1987;499:29–46. 3300488 10.1111/j.1749-6632.1987.tb36195.x

[34] BaHammam A, Alrajeh M, Albabtain M, Bahammam S, Sharif M. Circadian pattern of sleep, energy expenditure, and body temperature of young healthy men during the intermittent fasting of Ramadan. Appetite. 2010;54(2):426–9. 20100529 10.1016/j.appet.2010.01.011

[35] Alkandari JR, Maughan RJ, Roky R, Aziz AR, Karli U. The implications of Ramadan fasting for human health and well-being. J Sports Sci. 2012;30(Suppl 1):S9-19. 22742901 10.1080/02640414.2012.698298

[36] Trepanowski JF, Bloomer RJ. The impact of religious fasting on human health. Nutr J. 2010;9:57. 21092212 10.1186/1475-2891-9-57 PMC2995774

[37] Hepler C, Weidemann BJ, Waldeck NJ, Marcheva B, Cedernaes J, Thorne AK, et al. Time-restricted feeding mitigates obesity through adipocyte thermogenesis. Science. 2022;378(6617):276–84. 36264811 10.1126/science.abl8007 PMC10150371

[38] Damiola F, Le Minh N, Preitner N, Kornmann B, Fleury-Olela F, Schibler U. Restricted feeding uncouples circadian oscillators in peripheral tissues from the central pacemaker in the suprachiasmatic nucleus. Genes Dev. 2000;14(23):2950–61. 11114885 10.1101/gad.183500 PMC317100

[39] Hara R, Wan K, Wakamatsu H, Aida R, Moriya T, Akiyama M, et al. Restricted feeding entrains liver clock without participation of the suprachiasmatic nucleus. Genes Cells. 2001;6(3):269–78. 11260270 10.1046/j.1365-2443.2001.00419.x

[40] Kinouchi K, Magnan C, Ceglia N, Liu Y, Cervantes M, Pastore N, et al. Fasting Imparts a Switch to Alternative Daily Pathways in Liver and Muscle. Cell Rep. 2018;25(12):3299-314.e6. 30566858 10.1016/j.celrep.2018.11.077 PMC6433478

[41] Vollmers C, Gill S, DiTacchio L, Pulivarthy SR, Le HD, Panda S. Time of feeding and the intrinsic circadian clock drive rhythms in hepatic gene expression. Proc Natl Acad Sci U S A. 2009;106(50):21453–8. 19940241 10.1073/pnas.0909591106 PMC2795502

[42] Chaix A, Lin T, Le HD, Chang MW, Panda S. Time-Restricted Feeding Prevents Obesity and Metabolic Syndrome in Mice Lacking a Circadian Clock. Cell Metab. 2019;29(2):303-19.e4. 30174302 10.1016/j.cmet.2018.08.004 PMC7751278

[43] Cedernaes J, Huang W, Ramsey KM, Waldeck N, Cheng L, Marcheva B, et al. Transcriptional Basis for Rhythmic Control of Hunger and Metabolism within the AgRP Neuron. Cell Metab. 2019;29(5):1078-91.e5. 30827863 10.1016/j.cmet.2019.01.023 PMC6506361

[44] Mizuno TM, Makimura H, Silverstein J, Roberts JL, Lopingco T, Mobbs CV. Fasting regulates hypothalamic neuropeptide Y, agouti-related peptide, and proopiomelanocortin in diabetic mice independent of changes in leptin or insulin. Endocrinology. 1999;140(10):4551–7. 10499510 10.1210/endo.140.10.6966

[45] Hatori M, Vollmers C, Zarrinpar A, DiTacchio L, Bushong EA, Gill S, et al. Time-restricted feeding without reducing caloric intake prevents metabolic diseases in mice fed a high-fat diet. Cell Metab. 2012;15(6):848–60. 22608008 10.1016/j.cmet.2012.04.019 PMC3491655

[46] Lamia KA, Sachdeva UM, DiTacchio L, Williams EC, Alvarez JG, Egan DF, et al. AMPK regulates the circadian clock by cryptochrome phosphorylation and degradation. Science. 2009;326(5951):437–40. 19833968 10.1126/science.1172156 PMC2819106

[47] Kolbe I, Leinweber B, Brandenburger M, Oster H. Circadian clock network desynchrony promotes weight gain and alters glucose homeostasis in mice. Mol Metab. 2019;30:140–51. 31767165 10.1016/j.molmet.2019.09.012 PMC6807374

[48] Jordan SD, Lamia KA. AMPK at the crossroads of circadian clocks and metabolism. Mol Cell Endocrinol. 2013;366(2):163–9. 22750052 10.1016/j.mce.2012.06.017 PMC3502724

[49] Ramanathan C, Kathale ND, Liu D, Lee C, Freeman DA, Hogenesch JB, et al. mTOR signaling regulates central and peripheral circadian clock function. PLoS Genet. 2018;14(5):e1007369. 29750810 10.1371/journal.pgen.1007369 PMC5965903

[50] Khapre RV, Kondratova AA, Patel S, Dubrovsky Y, Wrobel M, Antoch MP, et al. BMAL1-dependent regulation of the mTOR signaling pathway delays aging. Aging (Albany NY). 2014;6(1):48–57. 24481314 10.18632/aging.100633 PMC3927809

[51] Neufeld TP. TOR-dependent control of autophagy: biting the hand that feeds. Curr Opin Cell Biol. 2010;22(2):157–68. 20006481 10.1016/j.ceb.2009.11.005 PMC2854204

[52] Brenna A, Ripperger JA, Saro G, Glauser DA, Yang Z, Albrecht U. PER2 mediates CREB-dependent light induction of the clock gene Per1. Sci Rep. 2021;11(1):21766. 34741086 10.1038/s41598-021-01178-6 PMC8571357

[53] Zhang EE, Liu Y, Dentin R, Pongsawakul PY, Liu AC, Hirota T, et al. Cryptochrome mediates circadian regulation of cAMP signaling and hepatic gluconeogenesis. Nat Med. 2010;16(10):1152–6. 20852621 10.1038/nm.2214 PMC2952072

[54] Shavlakadze T, Anwari T, Soffe Z, Cozens G, Mark PJ, Gondro C, et al. Impact of fasting on the rhythmic expression of myogenic and metabolic factors in skeletal muscle of adult mice. Am J Physiol Cell Physiol. 2013;305(1):C26-35. 23596176 10.1152/ajpcell.00027.2013

[55] Wullschleger S, Loewith R, Hall MN. TOR signaling in growth and metabolism. Cell. 2006;124(3):471–84. 16469695 10.1016/j.cell.2006.01.016

[56] de Goede P, Wüst RCI, Schomakers BV, Denis S, Vaz FM, Pras-Raves ML, et al. Time-restricted feeding during the inactive phase abolishes the daily rhythm in mitochondrial respiration in rat skeletal muscle. Faseb j. 2022;36(2):e22133. 35032416 10.1096/fj.202100707R

[57] Chapnik N, Genzer Y, Froy O. Relationship between FGF21 and UCP1 levels under time-restricted feeding and high-fat diet. J Nutr Biochem. 2017;40:116–21. 27883936 10.1016/j.jnutbio.2016.10.017

[58] Arredondo-Amador M, Zambrano C, Kulyté A, Luján J, Hu K, Sánchez de Medina F, et al. Circadian Rhythms in Hormone-sensitive Lipase in Human Adipose Tissue: Relationship to Meal Timing and Fasting Duration. J Clin Endocrinol Metab. 2020;105(12):e4407-16. 32725188 10.1210/clinem/dgaa492 PMC7538104

[59] Matoba K, Lu Y, Zhang R, Chen ER, Sangwung P, Wang B, et al. Adipose KLF15 Controls Lipid Handling to Adapt to Nutrient Availability. Cell Rep. 2017;21(11):3129–40. 29241541 10.1016/j.celrep.2017.11.032

[60] Couto Alves A, Glastonbury CA, El-Sayed Moustafa JS, Small KS. Fasting and time of day independently modulate circadian rhythm relevant gene expression in adipose and skin tissue. BMC Genomics. 2018;19(1):659. 30193568 10.1186/s12864-018-4997-y PMC6129005

[61] Brooks JF 2nd, Behrendt CL, Ruhn KA, Lee S, Raj P, Takahashi JS, et al. The microbiota coordinates diurnal rhythms in innate immunity with the circadian clock. Cell. 2021;184(16):4154-67.e12. 34324837 10.1016/j.cell.2021.07.001 PMC8967342

[62] Zarrinpar A, Chaix A, Yooseph S, Panda S. Diet and feeding pattern affect the diurnal dynamics of the gut microbiome. Cell Metab. 2014;20(6):1006–17. 25470548 10.1016/j.cmet.2014.11.008 PMC4255146

[63] Hernandez AR, Watson C, Federico QP, Fletcher R, Brotgandel A, Buford TW, et al. Twelve Months of Time-Restricted Feeding Improves Cognition and Alters Microbiome Composition Independent of Macronutrient Composition. Nutrients. 2022;14(19):3977. 10.3390/nu14193977 PMC9572159 36235630

[64] Zeb F, Wu X, Chen L, Fatima S, Ijaz Ul H, Chen A, et al. Time-restricted feeding is associated with changes in human gut microbiota related to nutrient intake. Nutrition. 2020;78:110797. 32540674 10.1016/j.nut.2020.110797

[65] Thaiss CA, Zeevi D, Levy M, Zilberman-Schapira G, Suez J, Tengeler AC, et al. Transkingdom control of microbiota diurnal oscillations promotes metabolic homeostasis. Cell. 2014;159(3):514–29. 25417104 10.1016/j.cell.2014.09.048

[66] Depner CM, Melanson EL, McHill AW, Wright KP Jr. Mistimed food intake and sleep alters 24-hour time-of-day patterns of the human plasma proteome. Proc Natl Acad Sci U S A. 2018;115(23):E5390–9. 29784788 10.1073/pnas.1714813115 PMC6003375

[67] Anothaisintawee T, Reutrakul S, Van Cauter E, Thakkinstian A. Sleep disturbances compared to traditional risk factors for diabetes development: Systematic review and meta-analysis. Sleep Med Rev. 2016;30:11–24. 26687279 10.1016/j.smrv.2015.10.002

[68] Lee Y, Seo E, Mun E, Lee W. A longitudinal study of working hours and chronic kidney disease in healthy workers: The Kangbuk Samsung Health Study. J Occup Health. 2021;63(1):e12266. 34382284 10.1002/1348-9585.12266 PMC8357818

[69] Torquati L, Mielke GI, Brown WJ, Kolbe-Alexander T. Shift work and the risk of cardiovascular disease. A systematic review and meta-analysis including dose-response relationship. Scand J Work Environ Health. 2018;44(3):229–38. 29247501 10.5271/sjweh.3700

[70] Rudic RD, McNamara P, Curtis AM, Boston RC, Panda S, Hogenesch JB, et al. BMAL1 and CLOCK, two essential components of the circadian clock, are involved in glucose homeostasis. PLoS Biol. 2004;2(11):e377. 15523558 10.1371/journal.pbio.0020377 PMC524471

[71] Morris CJ, Yang JN, Garcia JI, Myers S, Bozzi I, Wang W, et al. Endogenous circadian system and circadian misalignment impact glucose tolerance via separate mechanisms in humans. Proc Natl Acad Sci U S A. 2015;112(17):E2225–34. 25870289 10.1073/pnas.1418955112 PMC4418873

[72] Buxton OM, Cain SW, O’Connor SP, Porter JH, Duffy JF, Wang W, et al. Adverse metabolic consequences in humans of prolonged sleep restriction combined with circadian disruption. Sci Transl Med. 2012;4(129):129ra43. 22496545 10.1126/scitranslmed.3003200 PMC3678519

[73] Koritala BSC, Porter KI, Arshad OA, Gajula RP, Mitchell HD, Arman T, et al. Night shift schedule causes circadian dysregulation of DNA repair genes and elevated DNA damage in humans. J Pineal Res. 2021;70(3):e12726. 33638890 10.1111/jpi.12726 PMC8011353

[74] Salgado-Delgado R, Angeles-Castellanos M, Saderi N, Buijs RM, Escobar C. Food intake during the normal activity phase prevents obesity and circadian desynchrony in a rat model of night work. Endocrinology. 2010;151(3):1019–29. 20080873 10.1210/en.2009-0864

[75] Brown MR, Sen SK, Mazzone A, Her TK, Xiong Y, Lee JH, et al. Time-restricted feeding prevents deleterious metabolic effects of circadian disruption through epigenetic control of β cell function. Sci Adv. 2021;7(51):eabg6856. 34910509 10.1126/sciadv.abg6856 PMC8673777

[76] Mishra S, Persons PA, Lorenzo AM, Chaliki SS, Bersoux S. Time-restricted eating and its metabolic benefits. J Clin Med. 2023;12(22):7007. 10.3390/jcm12227007 PMC10672223 38002621

[77] Shabkhizan R, Haiaty S, Moslehian MS, Bazmani A, Sadeghsoltani F, Saghaei Bagheri H, et al. The Beneficial and Adverse Effects of Autophagic Response to Caloric Restriction and Fasting. Adv Nutr. 2023;14(5):1211–25. 37527766 10.1016/j.advnut.2023.07.006 PMC10509423

[78] Yorimitsu T, Klionsky DJ. Autophagy: molecular machinery for self-eating. Cell Death Differ. 2005;12(Suppl 2):1542–52. 16247502 10.1038/sj.cdd.4401765 PMC1828868

[79] Sun L, Xiong H, Chen L, Dai X, Yan X, Wu Y, et al. Deacetylation of ATG4B promotes autophagy initiation under starvation. Sci Adv. 2022;8(31):eabo0412. 35921421 10.1126/sciadv.abo0412 PMC9348796

[80] Mihaylova MM, Shaw RJ. The AMPK signalling pathway coordinates cell growth, autophagy and metabolism. Nat Cell Biol. 2011;13(9):1016–23. 21892142 10.1038/ncb2329 PMC3249400

[81] Kim J, Kim YC, Fang C, Russell RC, Kim JH, Fan W, et al. Differential regulation of distinct Vps34 complexes by AMPK in nutrient stress and autophagy. Cell. 2013;152(1–2):290–303. 23332761 10.1016/j.cell.2012.12.016 PMC3587159

[82] Kim YC, Guan KL. mTOR: a pharmacologic target for autophagy regulation. J Clin Invest. 2015;125(1):25–32. 25654547 10.1172/JCI73939 PMC4382265

[83] Martina JA, Chen Y, Gucek M, Puertollano R. MTORC1 functions as a transcriptional regulator of autophagy by preventing nuclear transport of TFEB. Autophagy. 2012;8(6):903–14. 22576015 10.4161/auto.19653 PMC3427256

[84] Ulgherait M, Midoun AM, Park SJ, Gatto JA, Tener SJ, Siewert J, et al. Circadian autophagy drives iTRF-mediated longevity. Nature. 2021;598(7880):353–8. 34588695 10.1038/s41586-021-03934-0 PMC9395244

[85] Yin Z, Klionsky DJ. Intermittent time-restricted feeding promotes longevity through circadian autophagy. Autophagy. 2022;18(3):471–2. 35220894 10.1080/15548627.2022.2039524 PMC9037462

[86] Jamshed H, Beyl RA, Della Manna DL, Yang ES, Ravussin E, Peterson CM. Early Time-Restricted Feeding Improves 24-Hour Glucose Levels and Affects Markers of the Circadian Clock, Aging, and Autophagy in Humans. Nutrients. 2019;11(6):1234. 31151228 10.3390/nu11061234 PMC6627766

[87] Saini SK, Singh A, Saini M, Gonzalez-Freire M, Leeuwenburgh C, Anton SD. Time-Restricted Eating Regimen Differentially Affects Circulatory miRNA Expression in Older Overweight Adults. Nutrients. 2022;14(9):1843. 35565812 10.3390/nu14091843 PMC9100641

[88] Namkoong S, Cho CS, Semple I, Lee JH. Autophagy Dysregulation and Obesity-Associated Pathologies. Mol Cells. 2018;41(1):3–10. 29370691 10.14348/molcells.2018.2213 PMC5792710

[89] Brand MD, Nicholls DG. Assessing mitochondrial dysfunction in cells. Biochem J. 2011;435(2):297–312. 21726199 10.1042/BJ20110162 PMC3076726

[90] Theurey P, Rieusset J. Mitochondria-Associated Membranes Response to Nutrient Availability and Role in Metabolic Diseases. Trends Endocrinol Metab. 2017;28(1):32–45. 27670636 10.1016/j.tem.2016.09.002

[91] de Mello AH, Costa AB, Engel JDG, Rezin GT. Mitochondrial dysfunction in obesity. Life Sci. 2018;192:26–32. 29155300 10.1016/j.lfs.2017.11.019

[92] Liesa M, Shirihai OS. Mitochondrial dynamics in the regulation of nutrient utilization and energy expenditure. Cell Metab. 2013;17(4):491–506. 23562075 10.1016/j.cmet.2013.03.002 PMC5967396

[93] Cantó C, Auwerx J. AMP-activated protein kinase and its downstream transcriptional pathways. Cell Mol Life Sci. 2010;67(20):3407–23. 20640476 10.1007/s00018-010-0454-z PMC3622821

[94] Rahman S, Islam R. Mammalian Sirt1: insights on its biological functions. Cell Commun Signal. 2011;9:11. 21549004 10.1186/1478-811X-9-11 PMC3103488

[95] Webster BR, Scott I, Traba J, Han K, Sack MN. Regulation of autophagy and mitophagy by nutrient availability and acetylation. Biochim Biophys Acta. 2014;1841(4):525–34. 24525425 10.1016/j.bbalip.2014.02.001 PMC3969632

[96] Ruderman NB, Xu XJ, Nelson L, Cacicedo JM, Saha AK, Lan F, et al. AMPK and SIRT1: a long-standing partnership? Am J Physiol Endocrinol Metab. 2010;298(4):E751–60. 20103737 10.1152/ajpendo.00745.2009 PMC2853213

[97] Hirschey MD, Shimazu T, Goetzman E, Jing E, Schwer B, Lombard DB, et al. SIRT3 regulates mitochondrial fatty-acid oxidation by reversible enzyme deacetylation. Nature. 2010;464(7285):121–5. 20203611 10.1038/nature08778 PMC2841477

[98] Ahn BH, Kim HS, Song S, Lee IH, Liu J, Vassilopoulos A, et al. A role for the mitochondrial deacetylase Sirt3 in regulating energy homeostasis. Proc Natl Acad Sci U S A. 2008;105(38):14447–52. 18794531 10.1073/pnas.0803790105 PMC2567183

[99] Hafner AV, Dai J, Gomes AP, Xiao CY, Palmeira CM, Rosenzweig A, et al. Regulation of the mPTP by SIRT3-mediated deacetylation of CypD at lysine 166 suppresses age-related cardiac hypertrophy. Aging (Albany NY). 2010;2(12):914–23. 21212461 10.18632/aging.100252 PMC3034180

[100] Theurey P, Tubbs E, Vial G, Jacquemetton J, Bendridi N, Chauvin MA, et al. Mitochondria-associated endoplasmic reticulum membranes allow adaptation of mitochondrial metabolism to glucose availability in the liver. J Mol Cell Biol. 2016;8(2):129–43. 26892023 10.1093/jmcb/mjw004

[101] Ahumada-Castro U, Bustos G, Silva-Pavez E, Puebla-Huerta A, Lovy A, Cárdenas C. In the Right Place at the Right Time: Regulation of Cell Metabolism by IP3R-Mediated Inter-Organelle Ca(2+) Fluxes. Front Cell Dev Biol. 2021;9:629522. 33738285 10.3389/fcell.2021.629522 PMC7960657

[102] Zhang SS, Zhou S, Crowley-McHattan ZJ, Wang RY, Li JP. A review of the role of endo/sarcoplasmic reticulum-mitochondria Ca(2+) transport in diseases and skeletal muscle function. Int J Environ Res Public Health. 2021;18(8):3874. 10.3390/ijerph18083874 PMC8067840 33917091

[103] Albalawi SS, Aljabri A, Alshibani M, Al-Gayyar MM. The Involvement of Calcium Channels in the Endoplasmic Reticulum Membrane in Nonalcoholic Fatty Liver Disease Pathogenesis. Cureus. 2023;15(11):e49150. 38024063 10.7759/cureus.49150 PMC10663096

[104] Castro-Sepúlveda M, Morio B, Tuñón-Suárez M, Jannas-Vela S, Díaz-Castro F, Rieusset J, et al. The fasting-feeding metabolic transition regulates mitochondrial dynamics. Faseb J. 2021;35(10):e21891. 34569666 10.1096/fj.202100929R

[105] Miwa S, Kashyap S, Chini E, von Zglinicki T. Mitochondrial dysfunction in cell senescence and aging. J Clin Invest. 2022;132(13):e158447. 10.1172/JCI158447 PMC9246372 35775483

[106] Keizer HG, Brands R, Oosting RS, Seinen W. A comprehensive model for the biochemistry of ageing, senescence and longevity. Biogerontology. 2024;25(4):615–26. 38441836 10.1007/s10522-024-10097-8

[107] Wortel IMN, van der Meer LT, Kilberg MS, van Leeuwen FN. Surviving Stress: Modulation of ATF4-Mediated Stress Responses in Normal and Malignant Cells. Trends Endocrinol Metab. 2017;28(11):794–806. 28797581 10.1016/j.tem.2017.07.003 PMC5951684

[108] Koyanagi S, Hamdan AM, Horiguchi M, Kusunose N, Okamoto A, Matsunaga N, et al. cAMP-response element (CRE)-mediated transcription by activating transcription factor-4 (ATF4) is essential for circadian expression of the Period2 gene. J Biol Chem. 2011;286(37):32416–23. 21768648 10.1074/jbc.M111.258970 PMC3173173

[109] Li W, Li X, Miller RA. ATF4 activity: a common feature shared by many kinds of slow-aging mice. Aging Cell. 2014;13(6):1012–8. 25156122 10.1111/acel.12264 PMC4326926

[110] Quirós PM, Prado MA, Zamboni N, D’Amico D, Williams RW, Finley D, et al. Multi-omics analysis identifies ATF4 as a key regulator of the mitochondrial stress response in mammals. J Cell Biol. 2017;216(7):2027–45. 28566324 10.1083/jcb.201702058 PMC5496626

[111] Na K, Park YJ. Protein Restriction in Metabolic Health: Lessons from Rodent Models. Nutrients. 2024;16(2):229. 38257122 10.3390/nu16020229 PMC10819042

[112] Zhang Y, Xie Y, Berglund ED, Coate KC, He TT, Katafuchi T, et al. The starvation hormone, fibroblast growth factor-21, extends lifespan in mice. Elife. 2012;1:e00065. 23066506 10.7554/eLife.00065 PMC3466591

[113] Lee SH, Lee JH, Lee HY, Min KJ. Sirtuin signaling in cellular senescence and aging. BMB Rep. 2019;52(1):24–34. 30526767 10.5483/BMBRep.2019.52.1.290 PMC6386230

[114] Shaw E, Talwadekar M, Rashida Z, Mohan N, Acharya A, Khatri S, et al. Anabolic SIRT4 exerts retrograde control over TORC1 signaling by glutamine sparing in the mitochondria. Mol Cell Biol. 2020;40(2):e00212–19. 10.1128/MCB.00212-19 PMC6944470 31685549

[115] Asadi Shahmirzadi A, Edgar D, Liao CY, Hsu YM, Lucanic M, Asadi Shahmirzadi A, et al. Alpha-Ketoglutarate, an Endogenous Metabolite, Extends Lifespan and Compresses Morbidity in Aging Mice. Cell Metab. 2020;32(3):447-56.e6. 32877690 10.1016/j.cmet.2020.08.004 PMC8508957

[116] Wood JG, Schwer B, Wickremesinghe PC, Hartnett DA, Burhenn L, Garcia M, et al. Sirt4 is a mitochondrial regulator of metabolism and lifespan in Drosophila melanogaster. Proc Natl Acad Sci U S A. 2018;115(7):1564–9. 29378963 10.1073/pnas.1720673115 PMC5816209

[117] Someya S, Yu W, Hallows WC, Xu J, Vann JM, Leeuwenburgh C, et al. Sirt3 mediates reduction of oxidative damage and prevention of age-related hearing loss under caloric restriction. Cell. 2010;143(5):802–12. 21094524 10.1016/j.cell.2010.10.002 PMC3018849

[118] Traba J, Geiger SS, Kwarteng-Siaw M, Han K, Ra OH, Siegel RM, et al. Prolonged fasting suppresses mitochondrial NLRP3 inflammasome assembly and activation via SIRT3-mediated activation of superoxide dismutase 2. J Biol Chem. 2017;292(29):12153–64. 28584055 10.1074/jbc.M117.791715 PMC5519366

[119] Garten A, Schuster S, Penke M, Gorski T, de Giorgis T, Kiess W. Physiological and pathophysiological roles of NAMPT and NAD metabolism. Nat Rev Endocrinol. 2015;11(9):535–46. 26215259 10.1038/nrendo.2015.117

[120] Nakahata Y, Sahar S, Astarita G, Kaluzova M, Sassone-Corsi P. Circadian control of the NAD+ salvage pathway by CLOCK-SIRT1. Science. 2009;324(5927):654–7. 19286518 10.1126/science.1170803 PMC6501775

[121] Ramsey KM, Yoshino J, Brace CS, Abrassart D, Kobayashi Y, Marcheva B, et al. Circadian clock feedback cycle through NAMPT-mediated NAD+ biosynthesis. Science. 2009;324(5927):651–4. 19299583 10.1126/science.1171641 PMC2738420

[122] Imai S, Guarente L. NAD+ and sirtuins in aging and disease. Trends Cell Biol. 2014;24(8):464–71. 24786309 10.1016/j.tcb.2014.04.002 PMC4112140

[123] Yang H, Yang T, Baur JA, Perez E, Matsui T, Carmona JJ, et al. Nutrient-sensitive mitochondrial NAD+ levels dictate cell survival. Cell. 2007;130(6):1095–107. 17889652 10.1016/j.cell.2007.07.035 PMC3366687

[124] Dahl TB, Holm S, Aukrust P, Halvorsen B. Visfatin/NAMPT: a multifaceted molecule with diverse roles in physiology and pathophysiology. Annu Rev Nutr. 2012;32:229–43. 22462624 10.1146/annurev-nutr-071811-150746

[125] Monteiro R, Azevedo I. Chronic inflammation in obesity and the metabolic syndrome. Mediators Inflamm. 2010;2010:289645. 10.1155/2010/289645 PMC2913796 20706689

[126] Kawai T, Autieri MV, Scalia R. Adipose tissue inflammation and metabolic dysfunction in obesity. Am J Physiol Cell Physiol. 2021;320(3):C375–91. 33356944 10.1152/ajpcell.00379.2020 PMC8294624

[127] Vandanmagsar B, Youm YH, Ravussin A, Galgani JE, Stadler K, Mynatt RL, et al. The NLRP3 inflammasome instigates obesity-induced inflammation and insulin resistance. Nat Med. 2011;17(2):179–88. 21217695 10.1038/nm.2279 PMC3076025

[128] Henao-Mejia J, Elinav E, Strowig T, Flavell RA. Inflammasomes: far beyond inflammation. Nat Immunol. 2012;13(4):321–4. 22430784 10.1038/ni.2257

[129] Traba J, Sack MN. The role of caloric load and mitochondrial homeostasis in the regulation of the NLRP3 inflammasome. Cell Mol Life Sci. 2017;74(10):1777–91. 27942750 10.1007/s00018-016-2431-7 PMC5391300

[130] Jager J, Grémeaux T, Cormont M, Le Marchand-Brustel Y, Tanti JF. Interleukin-1beta-induced insulin resistance in adipocytes through down-regulation of insulin receptor substrate-1 expression. Endocrinology. 2007;148(1):241–51. 17038556 10.1210/en.2006-0692 PMC1971114

[131] Litwiniuk A, Bik W, Kalisz M, Baranowska-Bik A. Inflammasome NLRP3 potentially links obesity-associated low-grade systemic inflammation and insulin resistance with alzheimer's disease. Int J Mol Sci. 2021;22(11):5603. 10.3390/ijms22115603 PMC8198882 34070553

[132] Traba J, Kwarteng-Siaw M, Okoli TC, Li J, Huffstutler RD, Bray A, et al. Fasting and refeeding differentially regulate NLRP3 inflammasome activation in human subjects. J Clin Invest. 2015;125(12):4592–600. 26529255 10.1172/JCI83260 PMC4665779

[133] Stienstra R, van Diepen JA, Tack CJ, Zaki MH, van de Veerdonk FL, Perera D, et al. Inflammasome is a central player in the induction of obesity and insulin resistance. Proc Natl Acad Sci U S A. 2011;108(37):15324–9. 21876127 10.1073/pnas.1100255108 PMC3174591

[134] Kim Y, Wang W, Okla M, Kang I, Moreau R, Chung S. Suppression of NLRP3 inflammasome by γ-tocotrienol ameliorates type 2 diabetes. J Lipid Res. 2016;57(1):66–76. 26628639 10.1194/jlr.M062828 PMC4689338

[135] Liang BJ, Liao SR, Huang WX, Huang C, Liu HS, Shen WZ. Intermittent fasting therapy promotes insulin sensitivity by inhibiting NLRP3 inflammasome in rat model. Ann Palliat Med. 2021;10(5):5299–309. 34107698 10.21037/apm-20-2410

[136] Liu B, Mao X, Huang D, Li F, Dong N. Novel role of NLRP3-inflammasome in regulation of lipogenesis in fasting-induced hepatic steatosis. Diabetes Metab Syndr Obes. 2019;12:801–11. 31239738 10.2147/DMSO.S206558 PMC6551611

[137] Piotrowska K, Zgutka K, Tomasiak P, Tarnowski M, Pawlik A. Every-other day (EOD) feeding regime decreases oxidative stress and inflammatory cascade in mouse liver: The immunohistochemical study. Tissue Cell. 2023;85:102236. 37812950 10.1016/j.tice.2023.102236

[138] Fann DY, Santro T, Manzanero S, Widiapradja A, Cheng YL, Lee SY, et al. Intermittent fasting attenuates inflammasome activity in ischemic stroke. Exp Neurol. 2014;257:114–9. 24805069 10.1016/j.expneurol.2014.04.017

[139] Poh L, Rajeev V, Selvaraji S, Lai MKP, Chen CL, Arumugam TV, et al. Intermittent fasting attenuates inflammasome-associated apoptotic and pyroptotic death in the brain following chronic hypoperfusion. Neurochem Int. 2021;148:105109. 34174333 10.1016/j.neuint.2021.105109

[140] Zeb F, Wu X, Fatima S, Zaman MH, Khan SA, Safdar M, et al. Time-restricted feeding regulates molecular mechanisms with involvement of circadian rhythm to prevent metabolic diseases. Nutrition. 2021;89:111244. 33930788 10.1016/j.nut.2021.111244

[141] Kersten S. The impact of fasting on adipose tissue metabolism. Biochim Biophys Acta Mol Cell Biol Lipids. 2023;1868(3):159262. 36521736 10.1016/j.bbalip.2022.159262

[142] Ibrahim M, Ayoub D, Wasselin T, Van Dorsselaer A, Le Maho Y, Raclot T, et al. Alterations in rat adipose tissue transcriptome and proteome in response to prolonged fasting. Biol Chem. 2020;401(3):389–405. 31398141 10.1515/hsz-2019-0184

[143] Vujović N, Piron MJ, Qian J, Chellappa SL, Nedeltcheva A, Barr D, et al. Late isocaloric eating increases hunger, decreases energy expenditure, and modifies metabolic pathways in adults with overweight and obesity. Cell Metab. 2022;34(10):1486-98.e7. 36198293 10.1016/j.cmet.2022.09.007 PMC10184753

[144] Crosby P, Hamnett R, Putker M, Hoyle NP, Reed M, Karam CJ, et al. Insulin/IGF-1 Drives PERIOD Synthesis to Entrain Circadian Rhythms with Feeding Time. Cell. 2019;177(4):896-909.e20. 31030999 10.1016/j.cell.2019.02.017 PMC6506277

[145] Goldbraikh D, Neufeld D, Eid-Mutlak Y, Lasry I, Gilda JE, Parnis A, et al. USP1 deubiquitinates Akt to inhibit PI3K-Akt-FoxO signaling in muscle during prolonged starvation. EMBO Rep. 2020;21(4):e48791. 32133736 10.15252/embr.201948791 PMC7132338

[146] Rojas FA, Hirata AE, Saad MJ. Regulation of IRS-2 tyrosine phosphorylation in fasting and diabetes. Mol Cell Endocrinol. 2001;183(1–2):63–9. 11604226 10.1016/s0303-7207(01)00597-4

[147] McGuire CM, Forgac M. Glucose starvation increases V-ATPase assembly and activity in mammalian cells through AMP kinase and phosphatidylinositide 3-kinase/Akt signaling. J Biol Chem. 2018;293(23):9113–23. 29540478 10.1074/jbc.RA117.001327 PMC5995505

[148] Wang N, Ren L, Danser AHJ. Vacuolar H(+)-ATPase in Diabetes, Hypertension, and Atherosclerosis. Microcirculation. 2024;31(5):e12855. 38683673 10.1111/micc.12855

[149] Eberlé D, Hegarty B, Bossard P, Ferré P, Foufelle F. SREBP transcription factors: master regulators of lipid homeostasis. Biochimie. 2004;86(11):839–48. 15589694 10.1016/j.biochi.2004.09.018

[150] Agbonifo-Chijiokwu E, Nwangwa KE, Oyovwi MO, Ben-Azu B, Naiho AO, Emojevwe V, et al. Underlying biochemical effects of intermittent fasting, exercise and honey on streptozotocin-induced liver damage in rats. J Diabetes Metab Disord. 2023;22(1):515–27. 37255765 10.1007/s40200-022-01173-2 PMC10225416

[151] Lee JH, Kang HS, Park HY, Moon YA, Kang YN, Oh BC, et al. PPARα-dependent Insig2a overexpression inhibits SREBP-1c processing during fasting. Sci Rep. 2017;7(1):9958. 28855656 10.1038/s41598-017-10523-7 PMC5577246

[152] Tsintzas K, Jewell K, Kamran M, Laithwaite D, Boonsong T, Littlewood J, et al. Differential regulation of metabolic genes in skeletal muscle during starvation and refeeding in humans. J Physiol. 2006;575(Pt 1):291–303. 16763003 10.1113/jphysiol.2006.109892 PMC1819428

[153] Chowdhury MAR, An J, Jeong S. The Pleiotropic Face of CREB Family Transcription Factors. Mol Cells. 2023;46(7):399–413. 37013623 10.14348/molcells.2023.2193 PMC10336275

[154] Besse-Patin A, Jeromson S, Levesque-Damphousse P, Secco B, Laplante M, Estall JL. PGC1A regulates the IRS1:IRS2 ratio during fasting to influence hepatic metabolism downstream of insulin. Proc Natl Acad Sci U S A. 2019;116(10):4285–90. 30770439 10.1073/pnas.1815150116 PMC6410797

[155] Gibson AA, Sainsbury A. Strategies to Improve Adherence to Dietary Weight Loss Interventions in Research and Real-World Settings. Behav Sci (Basel). 2017;7(3):44. 28696389 10.3390/bs7030044 PMC5618052

[156] Tacad DKM, Tovar AP, Richardson CE, Horn WF, Krishnan GP, Keim NL, et al. Satiety Associated with Calorie Restriction and Time-Restricted Feeding: Peripheral Hormones. Adv Nutr. 2022;13(3):792–820. 35191467 10.1093/advances/nmac014 PMC9156388

[157] Tacad DKM, Tovar AP, Richardson CE, Horn WF, Keim NL, Krishnan GP, et al. Satiety Associated with Calorie Restriction and Time-Restricted Feeding: Central Neuroendocrine Integration. Adv Nutr. 2022;13(3):758–91. 35134815 10.1093/advances/nmac011 PMC9156369

[158] Druce M, Bloom SR. The regulation of appetite. Arch Dis Child. 2006;91(2):183–7. 16428368 10.1136/adc.2005.073759 PMC2082685

[159] Sohn JW. Network of hypothalamic neurons that control appetite. BMB Rep. 2015;48(4):229–33. 25560696 10.5483/BMBRep.2015.48.4.272 PMC4436859

[160] Wynne K, Stanley S, McGowan B, Bloom S. Appetite control. J Endocrinol. 2005;184(2):291–318. 15684339 10.1677/joe.1.05866

[161] Gotthardt JD, Verpeut JL, Yeomans BL, Yang JA, Yasrebi A, Roepke TA, et al. Intermittent Fasting Promotes Fat Loss With Lean Mass Retention, Increased Hypothalamic Norepinephrine Content, and Increased Neuropeptide Y Gene Expression in Diet-Induced Obese Male Mice. Endocrinology. 2016;157(2):679–91. 26653760 10.1210/en.2015-1622 PMC4733124

[162] Lauzurica N, García-García L, Pinto S, Fuentes JA, Delgado M. Changes in NPY and POMC, but not serotonin transporter, following a restricted feeding/repletion protocol in rats. Brain Res. 2010;1313:103–12. 19968967 10.1016/j.brainres.2009.11.075

[163] Spezani R, da Silva RR, Martins FF, de Souza MT, Aguila MB, Mandarim-de-Lacerda CA. Intermittent fasting, adipokines, insulin sensitivity, and hypothalamic neuropeptides in a dietary overload with high-fat or high-fructose diet in mice. J Nutr Biochem. 2020;83:108419. 32580132 10.1016/j.jnutbio.2020.108419

[164] Zhang LN, Mitchell SE, Hambly C, Morgan DG, Clapham JC, Speakman JR. Physiological and behavioral responses to intermittent starvation in C57BL/6J mice. Physiol Behav. 2012;105(2):376–87. 21907222 10.1016/j.physbeh.2011.08.035

[165] Oliveira LDC, Morais GP, de Oliveira FP, Mata MM, Veras ASC, da Rocha AL, et al. Intermittent fasting combined with exercise training reduces body mass and alleviates hypothalamic disorders induced by high-fat diet intake. J Nutr Biochem. 2023;119:109372. 37169229 10.1016/j.jnutbio.2023.109372

[166] Yoshihara T, Honma S, Honma K. Effects of restricted daily feeding on neuropeptide Y release in the rat paraventricular nucleus. Am J Physiol. 1996;270(4 Pt 1):E589–95. 8928763 10.1152/ajpendo.1996.270.4.E589

[167] Sorrell J, Yates E, Rivir M, Woods SC, Hogenesch JB, Perez-Tilve D. The central melanocortin system mediates the benefits of time-restricted feeding on energy balance. Physiol Behav. 2020;227:113132. 32791179 10.1016/j.physbeh.2020.113132

[168] Wang D, Opperhuizen AL, Reznick J, Turner N, Su Y, Cooney GJ, et al. Effects of feeding time on daily rhythms of neuropeptide and clock gene expression in the rat hypothalamus. Brain Res. 2017;1671:93–101. 28709906 10.1016/j.brainres.2017.07.006

[169] Chaput JP, McHill AW, Cox RC, Broussard JL, Dutil C, da Costa BGG, et al. The role of insufficient sleep and circadian misalignment in obesity. Nat Rev Endocrinol. 2023;19(2):82–97. 36280789 10.1038/s41574-022-00747-7 PMC9590398

[170] Briggs DI, Andrews ZB. Metabolic status regulates ghrelin function on energy homeostasis. Neuroendocrinology. 2011;93(1):48–57. 21124019 10.1159/000322589

[171] Olszanecka-Glinianowicz M, Zahorska-Markiewicz B, Kocełak P, Janowska J, Semik-Grabarczyk E. The effect of weight reduction on plasma concentrations of ghrelin and insulin-like growth factor 1 in obese women. Endokrynol Pol. 2008;59(4):301–4. 18777499

[172] Pfluger PT, Kampe J, Castaneda TR, Vahl T, D’Alessio DA, Kruthaupt T, et al. Effect of human body weight changes on circulating levels of peptide YY and peptide YY3-36. J Clin Endocrinol Metab. 2007;92(2):583–8. 17119001 10.1210/jc.2006-1425

[173] Hayes MR, Mietlicki-Baase EG, Kanoski SE, De Jonghe BC. Incretins and amylin: neuroendocrine communication between the gut, pancreas, and brain in control of food intake and blood glucose. Annu Rev Nutr. 2014;34:237–60. 24819325 10.1146/annurev-nutr-071812-161201 PMC4458367

[174] Harvie MN, Pegington M, Mattson MP, Frystyk J, Dillon B, Evans G, et al. The effects of intermittent or continuous energy restriction on weight loss and metabolic disease risk markers: a randomized trial in young overweight women. Int J Obes (Lond). 2011;35(5):714–27. 20921964 10.1038/ijo.2010.171 PMC3017674

[175] Pureza I, Macena ML, da Silva Junior AE, Praxedes DRS, Vasconcelos LGL, Bueno NB. Effect of early time-restricted feeding on the metabolic profile of adults with excess weight: A systematic review with meta-analysis. Clin Nutr. 2021;40(4):1788–99. 33139084 10.1016/j.clnu.2020.10.031

[176] Zheng D, Liu S, Cabeza de Vaca S, Carr KD. Effects of time of feeding on psychostimulant reward, conditioned place preference, metabolic hormone levels, and nucleus accumbens biochemical measures in food-restricted rats. Psychopharmacology (Berl). 2013;227(2):307–20. 23354537 10.1007/s00213-013-2981-4 PMC3637844

[177] Sundaram S, Yan L. Time-restricted feeding reduces adiposity in mice fed a high-fat diet. Nutr Res. 2016;36(6):603–11. 27188906 10.1016/j.nutres.2016.02.005

[178] Ravussin E, Beyl RA, Poggiogalle E, Hsia DS, Peterson CM. Early Time-Restricted Feeding Reduces Appetite and Increases Fat Oxidation But Does Not Affect Energy Expenditure in Humans. Obesity (Silver Spring). 2019;27(8):1244–54. 31339000 10.1002/oby.22518 PMC6658129

[179] Hutchison AT, Regmi P, Manoogian ENC, Fleischer JG, Wittert GA, Panda S, et al. Time-Restricted Feeding Improves Glucose Tolerance in Men at Risk for Type 2 Diabetes: A Randomized Crossover Trial. Obesity (Silver Spring). 2019;27(5):724–32. 31002478 10.1002/oby.22449

[180] Jakubowicz D, Barnea M, Wainstein J, Froy O. High caloric intake at breakfast vs dinner differentially influences weight loss of overweight and obese women. Obesity (Silver Spring). 2013;21(12):2504–12. 23512957 10.1002/oby.20460

[181] Al-Rawi N, Madkour M, Jahrami H, Salahat D, Alhasan F, BaHammam A, et al. Effect of diurnal intermittent fasting during Ramadan on ghrelin, leptin, melatonin, and cortisol levels among overweight and obese subjects: A prospective observational study. PLoS ONE. 2020;15(8):e0237922. 32845924 10.1371/journal.pone.0237922 PMC7449475

[182] Zouhal H, Bagheri R, Triki R, Saeidi A, Wong A, Hackney AC, et al. Effects of ramadan intermittent fasting on gut hormones and body composition in males with obesity. Int J Environ Res Public Health. 2020;17(15):5600. 10.3390/ijerph17155600 PMC7432640 32756479

[183] Alzoghaibi MA, Pandi-Perumal SR, Sharif MM, BaHammam AS. Diurnal intermittent fasting during Ramadan: the effects on leptin and ghrelin levels. PLoS ONE. 2014;9(3):e92214. 24637892 10.1371/journal.pone.0092214 PMC3956913

[184] Carlson O, Martin B, Stote KS, Golden E, Maudsley S, Najjar SS, et al. Impact of reduced meal frequency without caloric restriction on glucose regulation in healthy, normal-weight middle-aged men and women. Metabolism. 2007;56(12):1729–34. 17998028 10.1016/j.metabol.2007.07.018 PMC2121099

[185] Allison KC, Hopkins CM, Ruggieri M, Spaeth AM, Ahima RS, Zhang Z, et al. Prolonged, Controlled Daytime versus Delayed Eating Impacts Weight and Metabolism. Curr Biol. 2021;31(3):650-7.e3. 33259790 10.1016/j.cub.2020.10.092 PMC7878354

[186] Hoddy KK, Gibbons C, Kroeger CM, Trepanowski JF, Barnosky A, Bhutani S, et al. Changes in hunger and fullness in relation to gut peptides before and after 8 weeks of alternate day fasting. Clin Nutr. 2016;35(6):1380–5. 27062219 10.1016/j.clnu.2016.03.011

[187] Sutton EF, Beyl R, Early KS, Cefalu WT, Ravussin E, Peterson CM. Early Time-Restricted Feeding Improves Insulin Sensitivity, Blood Pressure, and Oxidative Stress Even without Weight Loss in Men with Prediabetes. Cell Metab. 2018;27(6):1212-21.e3. 29754952 10.1016/j.cmet.2018.04.010 PMC5990470

[188] Parr EB, Devlin BL, Radford BE, Hawley JA. A Delayed Morning and Earlier Evening Time-Restricted Feeding Protocol for Improving Glycemic Control and Dietary Adherence in Men with Overweight/Obesity: A Randomized Controlled Trial. Nutrients. 2020;12(2):505. 32079327 10.3390/nu12020505 PMC7071240

[189] Meneguetti BT, Cardoso MH, Ribeiro CFA, Felício MR, Pinto IB, Santos NC, et al. Neuropeptide receptors as potential pharmacological targets for obesity. Pharmacol Ther. 2019;196:59–78. 30439454 10.1016/j.pharmthera.2018.11.002

[190] Hay DL, Chen S, Lutz TA, Parkes DG, Roth JD. Amylin: Pharmacology, Physiology, and Clinical Potential. Pharmacol Rev. 2015;67(3):564–600. 26071095 10.1124/pr.115.010629

[191] Boucher J, Kleinridders A, Kahn CR. Insulin receptor signaling in normal and insulin-resistant states. Cold Spring Harb Perspect Biol. 2014;6(1):a009191. 10.1101/cshperspect.a009191 PMC3941218 24384568

[192] Ahmed B, Sultana R, Greene MW. Adipose tissue and insulin resistance in obese. Biomed Pharmacother. 2021;137:111315. 33561645 10.1016/j.biopha.2021.111315

[193] Banks WA, Owen JB, Erickson MA. Insulin in the brain: there and back again. Pharmacol Ther. 2012;136(1):82–93. 22820012 10.1016/j.pharmthera.2012.07.006 PMC4134675

[194] Oktedalen O, Opstad PK, Waldum H, Jorde R. The fasting levels and the postprandial response of gastroenteropancreatic hormones before and after prolonged fasting. Scand J Gastroenterol. 1983;18(4):555–60. 6669932 10.3109/00365528309181637

[195] Gabel K, Hoddy KK, Haggerty N, Song J, Kroeger CM, Trepanowski JF, et al. Effects of 8-hour time restricted feeding on body weight and metabolic disease risk factors in obese adults: A pilot study. Nutr Healthy Aging. 2018;4(4):345–53. 29951594 10.3233/NHA-170036 PMC6004924

[196] Albosta M, Bakke J. Intermittent fasting: is there a role in the treatment of diabetes? A review of the literature and guide for primary care physicians. Clin Diabetes Endocrinol. 2021;7(1):3. 33531076 10.1186/s40842-020-00116-1 PMC7856758

[197] Barnosky AR, Hoddy KK, Unterman TG, Varady KA. Intermittent fasting vs daily calorie restriction for type 2 diabetes prevention: a review of human findings. Transl Res. 2014;164(4):302–11. 24993615 10.1016/j.trsl.2014.05.013

[198] Li C, Xing C, Zhang J, Zhao H, Shi W, He B. Eight-hour time-restricted feeding improves endocrine and metabolic profiles in women with anovulatory polycystic ovary syndrome. J Transl Med. 2021;19(1):148. 33849562 10.1186/s12967-021-02817-2 PMC8045367

[199] Che T, Yan C, Tian D, Zhang X, Liu X, Wu Z. Time-restricted feeding improves blood glucose and insulin sensitivity in overweight patients with type 2 diabetes: a randomised controlled trial. Nutr Metab (Lond). 2021;18(1):88. 34620199 10.1186/s12986-021-00613-9 PMC8499480

[200] Cienfuegos S, Gabel K, Kalam F, Ezpeleta M, Wiseman E, Pavlou V, et al. Effects of 4- and 6-h Time-Restricted Feeding on Weight and Cardiometabolic Health: A Randomized Controlled Trial in Adults with Obesity. Cell Metab. 2020;32(3):366-78.e3. 32673591 10.1016/j.cmet.2020.06.018 PMC9407646

[201] Kershaw EE, Flier JS. Adipose tissue as an endocrine organ. J Clin Endocrinol Metab. 2004;89(6):2548–56. 15181022 10.1210/jc.2004-0395

[202] Chouchani ET, Kajimura S. Metabolic adaptation and maladaptation in adipose tissue. Nat Metab. 2019;1(2):189–200. 31903450 10.1038/s42255-018-0021-8 PMC6941795

[203] Considine RV, Sinha MK, Heiman ML, Kriauciunas A, Stephens TW, Nyce MR, et al. Serum immunoreactive-leptin concentrations in normal-weight and obese humans. N Engl J Med. 1996;334(5):292–5. 8532024 10.1056/NEJM199602013340503

[204] Halberg N, Henriksen M, Söderhamn N, Stallknecht B, Ploug T, Schjerling P, et al. Effect of intermittent fasting and refeeding on insulin action in healthy men. J Appl Physiol (1985). 2005;99(6):2128–36. 16051710 10.1152/japplphysiol.00683.2005

[205] Finucane FM, Luan J, Wareham NJ, Sharp SJ, O’Rahilly S, Balkau B, et al. Correlation of the leptin:adiponectin ratio with measures of insulin resistance in non-diabetic individuals. Diabetologia. 2009;52(11):2345–9. 19756488 10.1007/s00125-009-1508-3 PMC2759015

[206] Heilbronn LK, Smith SR, Martin CK, Anton SD, Ravussin E. Alternate-day fasting in nonobese subjects: effects on body weight, body composition, and energy metabolism. Am J Clin Nutr. 2005;81(1):69–73. 15640462 10.1093/ajcn/81.1.69

[207] Cani PD. Human gut microbiome: hopes, threats and promises. Gut. 2018;67(9):1716–25. 29934437 10.1136/gutjnl-2018-316723 PMC6109275

[208] Mitev K, Taleski V. Association between the Gut Microbiota and Obesity. Open Access Maced J Med Sci. 2019;7(12):2050–6. 31406553 10.3889/oamjms.2019.586 PMC6684436

[209] Frank J, Gupta A, Osadchiy V, Mayer EA. Brain-gut-microbiome interactions and intermittent fasting in obesity. Nutrients. 2021;13(2):584. 10.3390/nu13020584 PMC7916460 33578763

[210] Zeb F, Osaili T, Obaid RS, Naja F, Radwan H, Cheikh Ismail L, et al. Gut Microbiota and time-restricted feeding/eating: a targeted biomarker and approach in precision nutrition. Nutrients. 2023;15(2):259. 10.3390/nu15020259 PMC9863108 36678130

[211] Wang Z, Koonen D, Hofker M, Fu J. Gut microbiome and lipid metabolism: from associations to mechanisms. Curr Opin Lipidol. 2016;27(3):216–24. 27054442 10.1097/MOL.0000000000000308

[212] Zhang L, Wang Y, Sun Y, Zhang X. Intermittent Fasting and Physical Exercise for Preventing Metabolic Disorders through Interaction with Gut Microbiota: A Review. Nutrients. 2023;15(10). 10.3390/nu15102277 PMC10224556 37242160

[213] Rust BM, Picklo MJ, Yan L, Mehus AA, Zeng H. Time-restricted feeding modifies the fecal lipidome and the gut microbiota. Nutrients. 2023;15(7):1562. 10.3390/nu15071562 PMC10096715 37049404

[214] Pinto FCS, Silva AAM, Souza SL. Repercussions of intermittent fasting on the intestinal microbiota community and body composition: a systematic review. Nutr Rev. 2022;80(3):613–28. 35020929 10.1093/nutrit/nuab108

[215] Popa AD, Niță O, Gherasim A, Enache AI, Caba L, Mihalache L, et al. A scoping review of the relationship between intermittent fasting and the human gut microbiota: current knowledge and future directions. Nutrients. 2023;15(9):2095. 10.3390/nu15092095 PMC10180719 37432222

[216] Guo Y, Luo S, Ye Y, Yin S, Fan J, Xia M. Intermittent Fasting Improves Cardiometabolic Risk Factors and Alters Gut Microbiota in Metabolic Syndrome Patients. J Clin Endocrinol Metab. 2021;106(1):64–79. 33017844 10.1210/clinem/dgaa644

[217] Mousavi SN, Rayyani E, Heshmati J, Tavasolian R, Rahimlou M. Effects of Ramadan and Non-ramadan Intermittent Fasting on Gut Microbiome. Front Nutr. 2022;9:860575. 35392284 10.3389/fnut.2022.860575 PMC8980861

[218] Zhang X, Zou Q, Zhao B, Zhang J, Zhao W, Li Y, et al. Effects of alternate-day fasting, time-restricted fasting and intermittent energy restriction DSS-induced on colitis and behavioral disorders. Redox Biol. 2020;32:101535. 32305005 10.1016/j.redox.2020.101535 PMC7162980

[219] Rinninella E, Cintoni M, Raoul P, Ianiro G, Laterza L, Lopetuso LR, et al. Gut microbiota during dietary restrictions: new insights in non-communicable diseases. Microorganisms. 2020;8(8):1140. 10.3390/microorganisms8081140 PMC7465033 32731505

[220] Shimada Y, Kinoshita M, Harada K, Mizutani M, Masahata K, Kayama H, et al. Commensal bacteria-dependent indole production enhances epithelial barrier function in the colon. PLoS ONE. 2013;8(11):e80604. 24278294 10.1371/journal.pone.0080604 PMC3835565

[221] Mohr AE, Jasbi P, Bowes DA, Dirks B, Whisner CM, Arciero KM, et al. Exploratory analysis of one versus two-day intermittent fasting protocols on the gut microbiome and plasma metabolome in adults with overweight/obesity. Front Nutr. 2022;9:1036080. 36386914 10.3389/fnut.2022.1036080 PMC9644216

[222] BaHammam AS, Pirzada A. Timing Matters: The Interplay between Early Mealtime, Circadian Rhythms, Gene Expression, Circadian Hormones, and Metabolism-A Narrative Review. Clocks Sleep. 2023;5(3):507–35. 37754352 10.3390/clockssleep5030034 PMC10528427

[223] Charlot A, Hutt F, Sabatier E, Zoll J. Beneficial effects of early time-restricted feeding on metabolic diseases: importance of aligning food habits with the circadian clock. Nutrients. 2021;13(5):1405. 10.3390/nu13051405 PMC8143522 33921979

[224] Espelund U, Hansen TK, Højlund K, Beck-Nielsen H, Clausen JT, Hansen BS, et al. Fasting unmasks a strong inverse association between ghrelin and cortisol in serum: studies in obese and normal-weight subjects. J Clin Endocrinol Metab. 2005;90(2):741–6. 15522942 10.1210/jc.2004-0604

[225] Boden G, Ruiz J, Urbain JL, Chen X. Evidence for a circadian rhythm of insulin secretion. Am J Physiol. 1996;271(2 Pt 1):E246–52. 8770017 10.1152/ajpendo.1996.271.2.E246

[226] Rynders CA, Morton SJ, Bessesen DH, Wright KP Jr, Broussard JL. Circadian Rhythm of Substrate Oxidation and Hormonal Regulators of Energy Balance. Obesity (Silver Spring). 2020;Suppl 1(Suppl 1):S104-s13. 10.1002/oby.22816 PMC7381359 32463976

[227] Ezpeleta M, Cienfuegos S, Lin S, Pavlou V, Gabel K, Tussing-Humphreys L, et al. Time-restricted eating: Watching the clock to treat obesity. Cell Metab. 2024;36(2):301–14. 38176412 10.1016/j.cmet.2023.12.004 PMC11221496

[228] Davis R, Rogers M, Coates AM, Leung GKW, Bonham MP. The Impact of Meal Timing on Risk of Weight Gain and Development of Obesity: a Review of the Current Evidence and Opportunities for Dietary Intervention. Curr Diab Rep. 2022;22(4):147–55. 35403984 10.1007/s11892-022-01457-0 PMC9010393

[229] Reid KJ, Baron KG, Zee PC. Meal timing influences daily caloric intake in healthy adults. Nutr Res. 2014;34(11):930–5. 25439026 10.1016/j.nutres.2014.09.010 PMC4794259

[230] Steger FL, Jamshed H, Martin CK, Richman JS, Bryan DR, Hanick CJ, et al. Impact of early time-restricted eating on diet quality, meal frequency, appetite, and eating behaviors: A randomized trial. Obesity (Silver Spring). 2023;Suppl 1(Suppl 1):127–38. 10.1002/oby.23642 PMC9945472 36575143

[231] Madjd A, Taylor MA, Delavari A, Malekzadeh R, Macdonald IA, Farshchi HR. Effects of consuming later evening meal v. earlier evening meal on weight loss during a weight loss diet: a randomised clinical trial. Br J Nutr. 2021;126(4):632–40. 33172509 10.1017/S0007114520004456

[232] Allison KC, Hopkins CM, Ruggieri M, Spaeth AM, Ahima RS, Zhang Z, et al. Prolonged, Controlled Daytime versus Delayed Eating Impacts Weight and Metabolism. Curr Biol. 2021;31(4):908. 33621495 10.1016/j.cub.2021.01.077 PMC7971800

[233] Maukonen M, Kanerva N, Partonen T, Kronholm E, Tapanainen H, Kontto J, et al. Chronotype differences in timing of energy and macronutrient intakes: A population-based study in adults. Obesity (Silver Spring). 2017;25(3):608–15. 28229553 10.1002/oby.21747

[234] Lombardo M, Bellia A, Padua E, Annino G, Guglielmi V, D’Adamo M, et al. Morning meal more efficient for fat loss in a 3-month lifestyle intervention. J Am Coll Nutr. 2014;33(3):198–205. 24809437 10.1080/07315724.2013.863169

[235] Dashti HS, Gómez-Abellán P, Qian J, Esteban A, Morales E, Scheer F, et al. Late eating is associated with cardiometabolic risk traits, obesogenic behaviors, and impaired weight loss. Am J Clin Nutr. 2021;113(1):154–61. 33022698 10.1093/ajcn/nqaa264 PMC7779221

[236] Lowe DA, Wu N, Rohdin-Bibby L, Moore AH, Kelly N, Liu YE, et al. Effects of Time-Restricted Eating on Weight Loss and Other Metabolic Parameters in Women and Men With Overweight and Obesity: The TREAT Randomized Clinical Trial. JAMA Intern Med. 2020;180(11):1491–9. 32986097 10.1001/jamainternmed.2020.4153 PMC7522780

[237] Schroder JD, Falqueto H, Mânica A, Zanini D, de Oliveira T, de Sá CA, et al. Effects of time-restricted feeding in weight loss, metabolic syndrome and cardiovascular risk in obese women. J Transl Med. 2021;19(1):3. 33407612 10.1186/s12967-020-02687-0 PMC7786967

[238] Przulj D, Ladmore D, Smith KM, Phillips-Waller A, Hajek P. Time restricted eating as a weight loss intervention in adults with obesity. PLoS ONE. 2021;16(1):e0246186. 33508009 10.1371/journal.pone.0246186 PMC7842957

[239] Kotarsky CJ, Johnson NR, Mahoney SJ, Mitchell SL, Schimek RL, Stastny SN, et al. Time-restricted eating and concurrent exercise training reduces fat mass and increases lean mass in overweight and obese adults. Physiol Rep. 2021;9(10):e14868. 34042299 10.14814/phy2.14868 PMC8157764

[240] Liu D, Huang Y, Huang C, Yang S, Wei X, Zhang P, et al. Calorie Restriction with or without Time-Restricted Eating in Weight Loss. N Engl J Med. 2022;386(16):1495–504. 35443107 10.1056/NEJMoa2114833

[241] Steger FL, Jamshed H, Bryan DR, Richman JS, Warriner AH, Hanick CJ, et al. Early time-restricted eating affects weight, metabolic health, mood, and sleep in adherent completers: A secondary analysis. Obesity (Silver Spring). 2023;Suppl 1(Suppl 1):96–107. 10.1002/oby.23614 PMC9877132 36518092

[242] Bantle AE, Lau KJ, Wang Q, Malaeb S, Harindhanavudhi T, Manoogian ENC, et al. Time-restricted eating did not alter insulin sensitivity or β-cell function in adults with obesity: A randomized pilot study. Obesity (Silver Spring). 2023;Suppl 1(Suppl 1):108–15. 10.1002/oby.23620 PMC9877119 36518093

[243] Parr EB, Kouw IWK, Wheeler MJ, Radford BE, Hall RC, Senden JM, et al. Eight-hour time-restricted eating does not lower daily myofibrillar protein synthesis rates: A randomized control trial. Obesity (Silver Spring). 2023;Suppl 1(Suppl 1):116–26. 10.1002/oby.23637 PMC10107304 36546330

[244] Wei X, Lin B, Huang Y, Yang S, Huang C, Shi L, et al. Effects of Time-Restricted Eating on Nonalcoholic Fatty Liver Disease: The TREATY-FLD Randomized Clinical Trial. JAMA Netw Open. 2023;6(3):e233513. 36930148 10.1001/jamanetworkopen.2023.3513 PMC10024204

[245] Lin S, Cienfuegos S, Ezpeleta M, Gabel K, Pavlou V, Mulas A, et al. Time-Restricted Eating Without Calorie Counting for Weight Loss in a Racially Diverse Population : A Randomized Controlled Trial. Ann Intern Med. 2023;176(7):885–95. 37364268 10.7326/M23-0052 PMC11192144

[246] Pavlou V, Cienfuegos S, Lin S, Ezpeleta M, Ready K, Corapi S, et al. Effect of Time-Restricted Eating on Weight Loss in Adults With Type 2 Diabetes: A Randomized Clinical Trial. JAMA Netw Open. 2023;6(10):e2339337. 37889487 10.1001/jamanetworkopen.2023.39337 PMC10611992

[247] Ameur R, Maaloul R, Tagougui S, Neffati F, Hadj Kacem F, Najjar MF, et al. Unlocking the power of synergy: High-intensity functional training and early time-restricted eating for transformative changes in body composition and cardiometabolic health in inactive women with obesity. PLoS ONE. 2024;19(5):e0301369. 38691521 10.1371/journal.pone.0301369 PMC11062533

[248] Cienfuegos S, Gabel K, Kalam F, Ezpeleta M, Pavlou V, Lin S, et al. The effect of 4-h versus 6-h time restricted feeding on sleep quality, duration, insomnia severity and obstructive sleep apnea in adults with obesity. Nutr Health. 2022;28(1):5–11. 33759620 10.1177/02601060211002347 PMC8460695

[249] Thomas EA, Zaman A, Sloggett KJ, Steinke S, Grau L, Catenacci VA, et al. Early time-restricted eating compared with daily caloric restriction: A randomized trial in adults with obesity. Obesity (Silver Spring). 2022;30(5):1027–38. 35470974 10.1002/oby.23420 PMC9046980

[250] Pureza I, Melo ISV, Macena ML, Praxedes DRS, Vasconcelos LGL, Silva-Júnior AE, et al. Acute effects of time-restricted feeding in low-income women with obesity placed on hypoenergetic diets: Randomized trial. Nutrition. 2020;77:110796. 32428840 10.1016/j.nut.2020.110796

[251] de Oliveira MaranhãoPureza IR, de Silva Junior AE, Silva Praxedes DR, LessaVasconcelos LG, de Lima Macena M, de Vieira Melo IS, et al. Effects of time-restricted feeding on body weight, body composition and vital signs in low-income women with obesity: A 12-month randomized clinical trial. Clin Nutr. 2021;40(3):759–66. 32713721 10.1016/j.clnu.2020.06.036

[252] He M, Wang J, Liang Q, Li M, Guo H, Wang Y, et al. Time-restricted eating with or without low-carbohydrate diet reduces visceral fat and improves metabolic syndrome: A randomized trial. Cell Rep Med. 2022;3(10):100777. 36220069 10.1016/j.xcrm.2022.100777 PMC9589024

[253] Maruthur NM, Pilla SJ, White K, Wu B, Maw MTT, Duan D, et al. Effect of Isocaloric, Time-Restricted Eating on Body Weight in Adults With Obesity : A Randomized Controlled Trial. Ann Intern Med. 2024;177(5):549–58. 38639542 10.7326/M23-3132 PMC13750091

[254] Quist JS, Pedersen HE, Jensen MM, Clemmensen KKB, Bjerre N, Ekblond TS, et al. Effects of 3 months of 10-h per-day time-restricted eating and 3 months of follow-up on bodyweight and cardiometabolic health in Danish individuals at high risk of type 2 diabetes: the RESET single-centre, parallel, superiority, open-label, randomised controlled trial. Lancet Healthy Longev. 2024;5(5):e314–25. 38588687 10.1016/S2666-7568(24)00028-X

[255] Haganes KL, Silva CP, Eyjólfsdóttir SK, Steen S, Grindberg M, Lydersen S, et al. Time-restricted eating and exercise training improve HbA1c and body composition in women with overweight/obesity: A randomized controlled trial. Cell Metab. 2022;34(10):1457-71.e4. 36198292 10.1016/j.cmet.2022.09.003

[256] Peeke PM, Greenway FL, Billes SK, Zhang D, Fujioka K. Effect of time restricted eating on body weight and fasting glucose in participants with obesity: results of a randomized, controlled, virtual clinical trial. Nutr Diabetes. 2021;11(1):6. 33446635 10.1038/s41387-021-00149-0 PMC7809455

[257] Termannsen AD, Varming A, van Elst C, Bjerre N, Nørgaard O, Hempler NF, et al. Feasibility of time-restricted eating in individuals with overweight, obesity, prediabetes, or type 2 diabetes: A systematic scoping review. Obesity (Silver Spring). 2023;31(6):1463–85. 37203334 10.1002/oby.23743

[258] Lao BN, Luo JH, Xu XY, Fu LZ, Tang F, Ouyang WW, et al. Time-restricted feeding’s effect on overweight and obese patients with chronic kidney disease stages 3–4: A prospective non-randomized control pilot study. Front Endocrinol (Lausanne). 2023;14:1096093. 37082115 10.3389/fendo.2023.1096093 PMC10111616

[259] O’Connor SG, Boyd P, Bailey CP, Nebeling L, Reedy J, Czajkowski SM, et al. A qualitative exploration of facilitators and barriers of adherence to time-restricted eating. Appetite. 2022;178:106266. 35934114 10.1016/j.appet.2022.106266 PMC9661403

[260] Fanaroff AC, Coratti S, Halaby R, Sanghavi M, O’Quinn RP, Krishnan S, et al. Feasibility and outcomes from using a commitment device and text message reminders to increase adherence to time-restricted eating: A randomized trial. Am Heart J. 2023;258:85–95. 36640862 10.1016/j.ahj.2022.12.010 PMC11010633

[261] Prasad M, Fine K, Gee A, Nair N, Popp CJ, Cheng B, et al. A smartphone intervention to promote time restricted eating reduces body weight and blood pressure in adults with overweight and obesity: a pilot study. Nutrients. 2021;13(7):2148. 10.3390/nu13072148 PMC8308240 34201442

[262] Wilkinson MJ, Manoogian ENC, Zadourian A, Lo H, Fakhouri S, Shoghi A, et al. Ten-Hour Time-Restricted Eating Reduces Weight, Blood Pressure, and Atherogenic Lipids in Patients with Metabolic Syndrome. Cell Metab. 2020;31(1):92-104.e5. 31813824 10.1016/j.cmet.2019.11.004 PMC6953486

[263] Zhao L, Hutchison AT, Liu B, Yates CL, Teong XT, Wittert GA, et al. Time-restricted eating improves glycemic control and dampens energy-consuming pathways in human adipose tissue. Nutrition. 2022;96:111583. 35150947 10.1016/j.nut.2021.111583

[264] Lin S, Cienfuegos S, Ezpeleta M, Pavlou V, Chakos K, McStay M, et al. Effect of time-restricted eating versus daily calorie restriction on mood and quality of life in adults with obesity. Nutrients. 2023;15(20):4313. 10.3390/nu15204313 PMC10609268 37892388

[265] Maaloul R, Marzougui H, Ben Dhia I, Ghroubi S, Tagougui S, Kallel C, et al. Effectiveness of Ramadan diurnal intermittent fasting and concurrent training in the management of obesity: is the combination worth the weight? Nutr Metab Cardiovasc Dis. 2023;33(3):659–66. 36710112 10.1016/j.numecd.2022.12.004

[266] Chan WK, Chuah KH, Rajaram RB, Lim LL, Ratnasingam J, Vethakkan SR. Metabolic Dysfunction-Associated Steatotic Liver Disease (MASLD): A State-of-the-Art Review. J Obes Metab Syndr. 2023;32(3):197–213. 37700494 10.7570/jomes23052 PMC10583766

[267] Kord-Varkaneh H, Salehi-Sahlabadi A, Tinsley GM, Santos HO, Hekmatdoost A. Effects of time-restricted feeding (16/8) combined with a low-sugar diet on the management of non-alcoholic fatty liver disease: A randomized controlled trial. Nutrition. 2023;105:111847. 36257081 10.1016/j.nut.2022.111847

[268] Deng Y, Liu X, Sun Y, Zhou L, Li Q, Lei Z, et al. Effects of time-restricted eating on intrahepatic fat and metabolic health among patients with nonalcoholic fatty liver disease. Obesity (Silver Spring). 2024;32(3):494–505. 38228496 10.1002/oby.23965

[269] Lange M, Nadkarni D, Martin L, Newberry C, Kumar S, Kushner T. Intermittent fasting improves hepatic end points in nonalcoholic fatty liver disease: A systematic review and meta-analysis. Hepatol Commun. 2023;7(8):e0212. 10.1097/HC9.0000000000000212 PMC10552959 37534936

