# Supplementary material for: Time Restricted Eating: A Valuable Alternative to Calorie Restriction for Addressing Obesity?
Source: Curr Obes Rep. 2025 Feb 3;14(1):17. doi: 10.1007/s13679-025-00609-z (PMC11790783; doi:10.1007/s13679-025-00609-z)
Supplement: Supplementary file 1 — Supplementary file1 (DOCX 30.9 KB) [file 13679_2025_609_MOESM1_ESM.docx]

**Supplementary table 1.** Summary of clinical trials investigating the effects of TRE on body weight and cardiovascular risk factors in adults affected by obesity.

| **Study reference** | **Intervention** | **Population** | **Most relevant findings** |
| --- | --- | --- | --- |
| Wilkinson et al, Cell Metab, 2020  [262] | Single-arm trial  10-h TRE  12 weeks | 19 participants  (6 females, 13 males)  Mean age 59 ± 11 years  Mean BMI 33 ± 5 kg/m^2^  Additional inclusion criteria: metabolic syndrome. | 1. Mean body weight -3%.   Mean waist circumference -4%.  Mean LDL-C -11%.  Non-significant improvement in HDL-C, fasting glucose, Hb1Ac.  No differences in triglycerides, hs-CRP, SBP and DBP. |
| Parr et al, Nutrients, 2020  [188] | Randomized, crossover  8-h TRE vs control  5 days | 11 participants  (11 males)  Mean age 38 ± 5 years  Mean BMI 32 ± 2 kg/m^2^ | 1. Non-significant improvement in 2. 24-h glycemic control. |
| De Oliveira Maranhão Pureza et al, Nutrition, 2020  [250] | Randomized  12-h TRE + CR vs CR  3 weeks | 58 participants  (58 females)  Mean age 32 ± 1 years  Mean BMI 31 ± 1 kg/m^2^ | 1. Mean body weight in TRE group: 2. -1.7%. 3. No significant between-group differences in weight loss, waist circumferences, SBP, DBP, fasting glucose. |
| De Oliveira Maranhão Pureza et al, Clin Nutr, 2021  [251] | Randomized  12-h TRE + CR vs CR  12 months | 58 participants  (58 females)  Mean age 32 ± 1 years  Mean BMI 31 ± 1 kg/m^2^ | (Per-protocol analysis, 13 TRE + CR vs 14 CR).   1. Mean body weight in TRE group 2. -0.9%.   No significant between-group differences in weight loss, waist circumferences, SBP, DBP, fasting glucose. |
| Lowe et al, JAMA Intern Med, 2020  [236] | Randomized  8-h TRE vs control  12 weeks | 116 participants  (46 females, 70 males)  Mean age 47 ± 1 years  Mean BMI 33 ± 1 kg/m^2^ | Mean body weight in TRE group  -1.8%.  No significant between-group differences in weight loss, waist circumference, SBP, DBP, fasting glucose, HbA1C, triglycerides, total cholesterol, LDL-C, HDL-C. |
| Schroder et al, J Transl Med, 2021  [237] | Non-randomized  8-h TRE vs control  12 weeks | 32 participants  (32 females)  Mean age 38 ± 1 years  Mean BMI 33 ± 1 kg/m^2^ | Significant reduction in body weight in TRE compared to controls (mean difference −4.9 kg).  No significant between-group differences in fasting glucose, SBP, DBP, LDL-C, HDL-C, triglycerides. |
| Peeke et al, Nutr Diabetes, 2021  [256] | Randomized  10-h TRE vs 12-h TRE  8 weeks | 60 participants  (53 females, 7 males)  Mean age 44 ± 1 years  Mean BMI 39 ± 1 kg/m^2^ | Significant weight loss compared to baseline (-8.5% and -7.1% for 10-hour TRE and 12-hour TRE, respectively).  Significant between group difference in body weight favoring the 10-h TRE group.  No significant between-group difference in fasting glucose. |
| Przulj et al, Plos One, 2021  [238] | Single-arm trial  8-h TRE  12 weeks | 51 participants  (37 females, 14 males)  Mean age 50 ± 2 years  Mean BMI 35 ± 1 kg/m^2^ | 1. Mean TRE adherence 5.1 days per week. 2. Mean weight loss -2.5 kg. 3. Participants who were adherent more than 5 days weekly lost more weight than those who did not.   No significant differences in LDL-C, SBP, DBP. |
| Cienfuegos et al, Nutr Health, 2021  [248] | Randomized  4-h TRE vs 6-h TRE vs control  8 weeks | 58 participants  (53 females, 5 males)  Mean age 47 ± 1 years  Mean BMI 37 ± 1 kg/m^2^ | Significant body weight reduction in 4-hour TRE group compared to control (mean difference -3.2%).  Significant weight reduction in 6-hour TRE group compared to control (mean difference -3.2%).  No significant between-group differences in fasting glucose, triglycerides, HDL-C, LDL-C. |
| Kotarsky et al, Physiol Rep, 2021  [239] | Randomized  8-h TRE vs control  Additional intervention for both groups: exercise training  8 weeks | 21 participants  (18 females, 3 males)  Mean age 44 ± 2 years  Mean BMI 30 ± 1 kg/m^2^ | 1. Significant body weight reduction in TRE group compared to control (mean difference -3.7%).   No significant between-group differences in Hb1Ac, HDL-C. |
| Prasad et al, Nutrients, 2021  [261] | Non-randomized  10-h TRE vs control  Additional intervention: TRE group participants received push notifications on their smartphones one hour before the beginning and the end of the prescribed eating window.  12 weeks | 50 participants  (41 females, 9 males)  Mean age 51 ± 3 years  Mean BMI 31 ± 3 kg/m^2^ | Mean body weight -1.6% in TRE group.  Mean waist circumference -2.0 cm in TRE group.  Mean SBP – 10 mmHg in TRE group.  No significant between-group differences in DBP. |
| Zhao et al, Nutrition, 2022  [263] | Single-arm trial  10-h TRE  8 weeks | 15 participants  (15 males)  Mean age 63 ± 4 years  Mean BMI 31 ± 2 kg/m^2^ | Mean body weight -2.4% from baseline.  Significant reduction in fasting glucose.  No significant changes in: SBP, DBP, triglycerides, HDL-C. |
| Liu et al, NEJM, 2022  [240] | Randomized  8-h TRE vs CR  12 months | 139 participants  (68 females, 71 males)  Mean age 32 ± 1 years  Mean BMI 31 ± 1 kg/m^2^ | Mean body weight -9.0% in TRE vs -7.2% in CR (no significant between-group difference).  No significant between-group differences in SBP, DBP, fasting glucose, LDL-C, HDL-C, triglycerides. |
| Thomas et al, Obesity, 2022  [249] | Randomized  E-TRE + CR vs CR  (E-TRE: 10-h TRE starting within 3 hours of waking up)  39 weeks | 81 participants  (69 females, 12 males)  Mean age 36 ± 6 years  Mean BMI 34 ± 1 kg/m^2^ | Mean body weight -5.3% in E-TRE group vs -4.3% in CR group (no significant between-group difference).  No significant between-group differences in Hb1Ac, LDL-C, HDL-C, triglycerides. |
| Haganes et al, Cell Metab, 2022  [255] | Randomized  10-h TRE vs HIIT vs 10-h TRE + HIIT vs  control  (HIIT: high intensity interval training)  7 weeks | 131 participants  (131 females)  Mean age 38 ± 1 years  Mean BMI 32 ± 1 kg/m^2^ | All interventions provide significant weigh loss compared to control, with no between-group differences: mean body weight -2.1 kg in TRE group vs -1.7 kg in HIIT group vs -3.6 kg in TRE+HIIT group vs -0.8 kg in control group.  TRE + HIIT significantly improved Hb1Ac and HDL-C compared to controls.  No significant between-group differences in LDL-C, triglycerides, SBP, DBP, fasting glucose. |
| He et al, Cell Met Rep, 2022  [252] | Randomized  TRE vs CR vs TRE + CR  12 weeks | 169 participants  (67 females, 102 males)  Mean age 41 ± 2 years  Mean BMI 30 ± 1 kg/m^2^ | TRE and TRE+CR induced significant weight loss compared to CR alone (-5.0 kg, -3.4 kg and -2.2 kg for TRE+CR, TRE and CR, respectively).  No significant between-group differences in LDL-C, HDL-C, triglycerides, SBP, DBP, fasting glucose and Hb1Ac. |
| Steger et al, Obesity, 2023  [241] | Randomized  8-h TRE + CR vs CR  14 weeks | 36 participants  (26 females, 10 males)  Mean age 50 ± 25 years  Mean BMI 38 ±6 kg/m^2^ | Mean body weight -7% in TRE+CR vs -4.1% in CR.  No significant between-group differences in: SBP, DBP, fasting insulin, HbA1c, total cholesterol, triglycerides, LDL-C and HDL-C. |
| Bantle et al, Obesity, 2023  [242] | Randomized  8-h TRE vs control  12 weeks | 20 participants  (17 females, 3 males)  Mean age 45 ± 12 years  Mean BMI 34 ±8 kg/m^2^ | No significant between-group differences in insulin resistance. |
| Parr et al, Obesity, 2023  [243] | Randomized  8-h TRE vs control  10 days | 18 participants  (18 males)  Mean age 46 ± 5 years  Mean BMI 30 ± 2 kg/m^2^ | 1. Non-significant improvement in 24-h glycemic control in TRE group.   No between-group differences in weight loss. |
| Fanaroff et al, Am Heart J, 2023  [260] | Randomized  Texting follow-up strategy vs commitment device  18 weeks | 37 participants  (22 females, 15 males)  Mean age 60 ± 11 years  Mean BMI 38 ±8 kg/m^2^ | No between-group differences in adherence in weight loss and blood pressure. |
| Maaloul et al, Nutr Metab Cardiovasc Dis, 2023  [265] | Randomized  RDIF vs RDIF + HIIT  (RDIF: Ramadan diurnal intermittent fasting, HIIT: high intensity interval training)  4 weeks | 20 participants  (20 males)  Mean age 32 ± 7 years  Mean BMI 33 ±4 kg/m^2^ | Both groups showed significant reduction in body weight, waist circumference and improvement in blood glucose and lipid profile.  RDIF + HIIT group showed greater improvement in lipid profile compared to RDIF group. |
| Wei et al, JAMA Netw Open, 2023  [244] | Randomized  8-h TRE vs control  6 months interventions +  6 months follow-up | 88 participants  (39 females, 49 males)  Mean age 32 ± 10 years  Mean BMI 32 ±3 kg/m^2^  Additional inclusion criteria: NAFLD. | No significant between-group differences in intrahepatic triglyceride content, liver stiffness and weight loss. |
| Lao et al, Front Endocrinol, 2023  [258] | Non-randomized  8-h TRE vs control  12 weeks | 28 participants  (14 females, 14 males)  Mean age 52 ± 10 years  Mean BMI 28 ± 3 kg/m^2^ | TRE improved eGRF, decreased acid uric and body weight.  No between-group differences in inflammatory factors. |
| Linn et al, Ann Intern Med, 2023  [245] | Randomized  8-h TRE vs CR vs control  12 months | 90 participants  (74 females, 16 males)    Mean age 44 ± 12 years  Mean BMI 38 ± 5 kg/m^2^ | No significant between-group differences in weight loss, SBP, DBP, fasting glucose, HbA1C, fasting glucose, fasting insulin, triglycerides, total cholesterol, LDL-C, HDL-C. |
| Pavlou et al, JAMA Netw Open, 2023  [246] | Randomized  8-h TRE vs CR vs control  6 months | 75 participants  (51 females, 24 males)  Mean age 55 ± 12 years  Mean BMI 39 ± 5 kg/m^2^  Additional inclusion criteria: type 2 diabetes. | Weight loss was significantly higher in TRE group vs control and CR.  TRE and CR improved HbA1c and fasting glucose compared to control. |
| Tricò et al, Diabetologia, 2024  [264] | Randomized  eTRC vs mediterranean diet  (eTRC: early time restricted carbohydrate consumption)  12 weeks | 23 participants  (11 females, 12 males)  Mean age 67 ± 8 years  Mean BMI 29 ± 4 kg/m^2^  Additional inclusion criteria: type 2 diabetes. | No between-groups differences in weight loss, HbA1c fasting plasma glucose, glucose tolerance, insulin resistance, insulin clearance and plasma glucagon levels. |
| Quist et al, Lancet Healthy Longev, 2024  [254] | Randomized  10-h TRE vs control  12 weeks | 100 participants  (66 females, 34 males)  Mean age 59 ± 6 years  Mean BMI 34 ± 6 kg/m^2^  Additional inclusion criteria: pre-diabetes if BMI between 25 and 30. | No significant difference in weight loss between TRE and control group (-1.2% vs -0.4% from baseline). |
| Maruthur et al, Ann Intern Med, 2024  [253] | Randomized  10-h isocaloric TRE vs control  12 weeks | 41 participants  (38 females, 3 males)  Mean age 59 years  Mean BMI 36 kg/m^2^ | No significant difference in weight loss between TRE and control group (-2.3 kg vs -2.6 kg from baseline).  No significant difference in glycemic measures. |
| Ameur et al, Plos One, 2024  [247] | Randomized  8-h TRE vs HIFT vs TRE + HIFT  (HIFT: high intensity functional training)  12 weeks | 64 participants  (64 females)  Mean age 32 ± 10 years  Mean BMI 35 ± 4 kg/m^2^ | TRE + HIFT group had superior effects on body weight, lipid profile, fasting glucose and systolic blood pressure compared to TRE alone or HIFT alone. |

Abbreviations: BMI, body mass index; CR, calorie restriction; TRE, time restricted eating. The search was conducted on PubMed on 30/JUN/2024; “time restricted eating”, “time restricted feeding” and “obesity” were the research items; research was limited to clinical trials; all abstracts were evaluated by 2 researchers (LC and MEP) and full text revision was performed for those articles who presented population (adults, BMI > 25 kg/m^2^) and outcomes (body weight, glycemic measures, lipid profile and blood pressure) considered pertinent for this review; in case of articles presenting secondary analysis on the same population, only the original study was included. When possible, values were rounded to the nearest whole number for simplification.
